# A Novel Regulation of K-antigen Capsule Synthesis in *Porphyromonas gingivalis* Is Driven by the Response Regulator PG0720-Directed Antisense RNA

**DOI:** 10.3389/froh.2021.701659

**Published:** 2021-07-01

**Authors:** Hey-Min Kim, Dev K. Ranjit, Alejandro R. Walker, Heran Getachew, Ann Progulske-Fox, Mary E. Davey

**Affiliations:** ^1^Department of Oral Biology, College of Dentistry, University of Florida, Gainesville, FL, United States; ^2^Department of Ophthalmology, Ocular Genomics Institute, Massachusetts Eye and Ear, Harvard Medical School, Boston, MA, United States

**Keywords:** response regulator PG0720, antisense RNA, surface polysaccharides, K-antigen capsule, *Porphyromonas gingivalis*

## Abstract

The periodontal pathogen *Porphyromonas gingivalis* strain W83 displays at least three different surface glycans, specifically two types of lipopolysaccharides (O-LPS and A-LPS) and K-antigen capsule. Despite the importance of K-antigen capsule to the virulence of *P. gingivalis*, little is known as to how expression of genes involved in the synthesis of this surface glycan is regulated. The genes required for K-antigen capsule synthesis are located in a locus that encodes a number of transcripts, including an operon (PG0104 to PG0121, generating ~19.4-kb transcript) which contains a non-coding 77-bp inverted repeat (77 bpIR) region near the 5'-end. Previously, we identified a 550-nucleotide antisense RNA molecule (designated asSuGR for antisense Surface Glycan Regulator) encoded within the 77-bpIR element that influences the synthesis of surface glycans. In this study, we demonstrate that the DNA-binding response regulator PG0720 can bind the promoter region of asSuGR and activate expression of asSuGR, indicating that PG0720 may indirectly influence transcript levels of the K-antigen capsule operon expressed from the sense strand. The data show that deletion of the PG0720 gene confers a defect in the presentation of surface polysaccharides compared with the parent strain and quantitative RT-PCR (qPCR) analysis determined that the overall expression of genes involved in K-antigen capsule synthesis were down-regulated in the PG0720 mutant. Furthermore, the defects of the PG0720 deletion mutant were restored by complementation. Importantly, the PG0720 deletion mutant showed reduced virulence. Altogether, our data show that the response regulator PG0720 regulates expression of asSuGR, a trans-acting antisense RNA molecule involved in modulating the production of surface polysaccharides in *P. gingivalis* strain W83. The data provide further evidence that surface glycans are key virulence determinants and significantly advances our understanding of the molecular mechanisms controlling the synthesis of *P. gingivalis* K-antigen capsule, a key virulence determinant.

## Introduction

Periodontal diseases are among the most common chronic biofilm-based infections of humans and are also associated with chronic systemic inflammatory disorders. Studies have shown that disease progression is linked to major changes in the gingival microenvironment caused by the host immune response [1] along with the function (metabolism and interactions) of indigenous microbiota [2–4]. The production of *Porphyromonas gingivalis* virulence factors, including proteases, adhesins, and surface glycans is influenced by its interactions with other oral bacteria and controlled by an elaborate signaling network of *P. gingivalis* based on serine/threonine and tyrosine phosphorylation/dephosphorylation [5, 6], extracytoplasmic function (ECF) sigma factors [7–10], and two-component systems (TCS) [11]. In general, TCS play an important role in bacterial sensing and responding to changes in the environmental cues. These systems consist of a sensor histidine kinase and a response regulator where an environmental signal activates the autophosphorylation of the histidine kinase on a conserved histidine within its sensor domain, then the high-energy phosphate is transferred to a conserved aspartate within the receiver domain of the cognate response regulator [12, 13]. The activated response regulator can then bind to the promoter regions of target genes to induce or repress gene expression. The genome of *P. gingivalis* strain W83 contains only four TCS pairs, along with one orphan histidine kinase, two orphan response regulators, and one chimeric TCS [14]. The focus of this study was on the response regulator PG0720 in strain W83. This response regulator is well-conserved in other strains of *P. gingivalis* as well as in *Bacteroides fragilis*. However, its cognate histidine kinase was found to be missing in *P. gingivalis* ATCC 33277. Transfer of PG0719 from strain W83 to ATCC 33277 restored growth defects [15].

Bacterial cell surface glycans influence host cell recognition and are considered key virulence determinants. *P. gingivalis* displays at least three different types of surface glycans such as O-lipopolysaccharides (O-LPS), A-lipopolysaccharides (A-LPS), and K-antigen capsule [16–19]. The surface glycans account for strain serotype specificity, specifically at least three different O-antigen serotypes and six K-antigen serotypes have been identified [20]. The K-antigen capsule of *P. gingivalis* strain W83 is associated with the severe form of infection, while infection with ATCC 33277 strain, which does not possess a capsule causes a localized abscess that does not disseminate in an animal infection model [21]. Bacterial encapsulation is known to protect pathogenic bacteria from clearance by host immune defenses [22] by masking the cell surface and thereby reducing the host's response [23]. Previously, our research group showed that the K-antigen locus encodes a large polycistronic transcript (PG0104 to PG0121) containing a 77-bp inverted repeat (77bpIR) region [24, 25]. Furthermore, a 550-nucleotide antisense RNA (designated asSuGR for antisense Surface Glycan Regulator) transcript produced from within the 77bpIR element was identified, demonstrating that transcription in the 77bpIR region is bidirectional [26]. Here, we further characterized asSuGR. Our data show that the DNA-binding response regulator PG0720 activates asSuGR expression, which in turn modulates the transcript level of genes within the K-antigen capsule locus located on the sense strand and the presentation of cell surface glycans.

## Materials and Methods

### Bacterial Strains and Mutant Construction

*P. gingivalis* strains were stored at −80°C and sub-cultured on blood agar plates (BAPHK) comprising Trypticase soy broth (Becton, Dickinson and Company, Franklin Lakes, NJ, USA), 5 μg/ml hemin, 1 μg/ml menadione, and 5% defibrinated sheep blood (Northeast Laboratory Services, Winslow, ME, USA) with incubation at 37°C in an anaerobic chamber (Coy Lab Products, Grass Lake, MI, USA) with an atmosphere containing 5% hydrogen, 10% carbon dioxide, and 85% nitrogen. Plates were grown anaerobically for 3–5 days. For liquid culture, the strains were grown in Trypticase soy broth (TSB) also supplemented with hemin and menadione (TSBHK). Strain W83 ΔPG0720 was generated as previously described [27]. Briefly, primers were designed to generate upstream and downstream products of ~1 kb flanking PG0720 as well as an erythromycin resistance gene (*ermF*) from plasmid pVA2198 [28]. Of note, the end of PG0720 overlaps with the start of PG0719, therefore a small portion (21 base pairs) of PG0720 was not deleted. All primers used in this study are presented in Supplementary Table 1. These oligonucleotides were used to amplify PCR products using genomic DNA (gDNA) and Phusion high-fidelity PCR master mix with HF buffer according to the manufacturer's instructions. The products were purified and combined using the NEBuilder HiFi DNA Assembly Master Mix (New England BioLabs, Ipswich, MA, USA) according to the instructions provided by the manufacturer. The final product was mixed with competent cells of *P. gingivalis* and transformed by electroporation as previously described [24]. The derivatives of *P. gingivalis* W83 were maintained by supplementing media with 10 μg/ml erythromycin or 1 μg/ml tetracycline. *Escherichia coli* strains were grown in Luria Broth (LB; Thermo Fisher Scientific) or on LB agar plates at 37°C. *E. coli* plasmid strains were maintained by supplementing the media with 100 μg/ml ampicillin. Details of bacterial strains and plasmid constructions are provided in the Supplementary Table 2.

### Electron Microscopy

Transmission electron microscopy and image analysis was performed by the electron microscopy core of Interdisciplinary Center for Biotechnology Research (ICBR) at the University of Florida. In order to visualize surface polysaccharides, colonies of *P. gingivalis* W83 and derivatives were scraped from blood agar plates and stained with ruthenium red as previously described [29].

### Preparation of Autoclaved Cell Extracts

*P. gingivalis* strain W83 and derivatives were inoculated into TSBHK and grown overnight. Cultures were diluted into fresh TSBHK, grown to mid-exponential phase and normalized to an optical density at 600 nm (OD_600_) of ~0.8. Cultures were diluted 1:5, and 10 μl aliquots of each culture were spotted on blood agar plates. After 4 days, cultures were scraped off the plates and placed into cuvettes containing sterile distilled water. Cultures were normalized to an OD_600_ of 1.0 in the final 1 ml and autoclaved at 120°C for 30 min. Once cooled, the extracts were centrifuged, and the supernatants were saved for analysis as previously described [27].

### Enzyme-Linked Immunosorbent Assay

Enzyme-linked immunosorbent assays (ELISAs) for the detection of surface polysaccharides were performed as previously described [26]. Briefly, autoclaved cell extracts (W83 and derivatives were diluted 1:1,000 in 50 mM carbonate/bicarbonate buffer, pH 9.6 and further serially diluted 2-fold in a 96-well microtiter plates (Microlon; Greiner Bio-One); plates containing diluted antigen were incubated at 4°C overnight. After washing plates with PBS containing 0.05% Tween 20 (PBS/Tween), a solution of 5% non-fat dry milk in PBS was used to block wells for ~1 h at room temperature. After washing with PBS/Tween, wells were incubated for 1 h at 37°C with a serotype specific antiserum previously generated against *P. gingivalis* strain W83 [24], diluted to a concentration of 1:2000 in PBS containing 0.1% Tween 20 and 0.1% bovine serum albumin (PTB), to detect the capsule. Wells were washed with PBS/Tween and then incubated for 1 h at 37°C with a goat anti-rabbit IgM-HRP antibody diluted at 1:5000 in PTB. To detect anionic polysaccharide (A-LPS), the monoclonal antibody 1B5 (kindly provided by Michael Curtis, Queen Mary University of London, London, England) was used at a concentration of 1:100, and an anti-mouse IgG-HPR antibody at 1:5000 was used as a secondary. After a final wash with PBS/Tween, wells were incubated with 3,3′,5,5′-Tetramethylbenzidine (Sigma–Aldrich, St. Louis, MO, USA) until sufficient color appeared (typically 20 min). The reaction was stopped with an equal portion of 1 M HCl, and the absorbance was recorded at OD_450_.

### Bacterial Growth

Broth cultures were grown anaerobically in Tryptic Soy Broth (TSB) medium (Becton, Dickinson and Company, Franklin Lakes, NJ, USA) supplemented with 5 μg/ml hemin and 1 μg/ml menadione (TSBHK). Overnight cultures were diluted 1:125 in pre-reduced TSBHK. Bacterial growths were then monitored by measuring the OD_600_ and presented as the mean ± standard deviations (*n* = 3).

### RNA Extraction, Sequencing, and qPCR Analysis

*P. gingivalis* strain W83 and its derivatives were inoculated in TSBHK and grown for overnight. The cultures were sub-cultured in pre-reduced TSBHK and grown to an OD_600_ of 0.8. Cultures were diluted 1:5 with fresh pre-reduced TSBHK, and 10 μl aliquots of each culture were spotted on blood agar plates. After 24 h incubation, cultures were scraped off the plates and the RNA extraction was performed using the Direct-zol RNA Miniprep kit (Zymo Research) according to the instructions provided by the manufacturer with a slight modification [29]. RNA samples were delivered to the Gene Expression and Genotyping core of Interdisciplinary Center for Biotechnology Research (ICBR) at the University of Florida. Sample quality determination and sequencing were performed by the Gene Expression and Genotyping core in the ICBR [30]. The bioinformatics section of this work was conducted entirely in the High Performance Cluster (HyperGator2) at the University of Florida. The quality control of the raw sequencing data, conducted with FastQC (Babraham Institute) revealed that the data had an average Phred score of 38, which according with our experience didn't require quality trimming and was parsed directly to Transabyss [31] for the short-reads transcriptome assembly. Subsequently we use the Burrows-Wheeler aligner [32] (BWA-mem) to map the ungapped reads to the reference genome *P. gingivalis* strain W83 (NCBI: NC_002950.2). Alignments files were then sorted with samtools [33] prior to counting transcriptomes mapped to reference genes with Htseq-counts [34]. The output files from Htseq-count were then parsed to R statistical program (https://www.r-project.org/) and analyzed with edgeR [35] in order to determine significant features. We eliminated any with a *p* > 0.05 and a fold change of <1.5. Raw sequencing data is available on the NCBI Sequence Read Archive (SRA) under accession number PRJNA725655. The qPCR was performed as described previously [36, 37]. Briefly, cDNA was produced from the same amount of RNA from each sample by using cDNA EcoDry Premix (Clontech). cDNAs were mixed with gene-specific primers and iQ SYBR Green Supermix (Bio-Rad). The qPCR was performed using the CFX96 Real-Time System (Bio-Rad).

### Computational Modeling of PG0720 for Identifying Phosphorylation and DNA Binding Sites

Alignment of response regulators with known crystal structures identified by I-TASSER [38, 39]. The sequences were obtained by NCBI database and software Expresso (tcoffee.crg.cat) was used to align them. Pymol version 2.3.2 was used to render and capture 90° rotational 3D structure.

### Protein Purification

To facilitate purification and removal of the purification tag, PG0720 was cloned into the *E. coli* expression vector pRham N-His SUMO (Lucigen). The primer design and cloning protocols provided by the manufacturer were used. *E. coli* 10G Chemically Competent Cells (Lucigen) were transformed with plasmids, which allows for further protein induction. The clones were screened by PCR and sequenced to verify that no mutations were introduced.

LB broth cultures were amended with 50 μg kanamycin ml^−1^ and inoculated with *E. coli* containing pRham N-His SUMO-PG0720. Cultures were grown at 37°C, on a platform shaker at 250 rpm, when cultures reached an optical density at 600 nm (OD_600_) of 0.5, rhamnose was added to the culture at a final concentration of 0.05% to induce expression. After 4 h incubation at 37°C, cells were then pelleted at 4,255 *g* for 10 min and the supernatants were discarded. Pellets were frozen at −20°C. To purify the proteins, 3 ml lysis buffer (50 mM NaH_2_PO_4_, 300 mM NaCl, pH 8.0) per 1 g wet weight of the pellet was added and cells were then disrupted by sonication. Lysate was centrifuged at 15,000 *g* for 20 min at 4°C. Centrifuge columns (Pierce) were equilibrated with 1 ml washing buffer (50 mM NaH_2_PO_4_, 300 mM NaCl, 20 mM imidazole, pH 8.0); followed by addition of Ni-NTA-agarose (0.5 ml per 250 ml culture; HisPur Ni-NTA, ThermoFisher) to the column, which was then equilibrated with 5 ml washing buffer. Lysates were transferred to the column and incubated for 10 min on room temperature, with mixing. The agarose was washed five times with 2 ml washing buffer. Ni-bound protein was eluted with 2 ml elution buffer (50 mM NaH_2_PO_4_, 300 mM NaCl, 250 mM imidazole, pH 8.0). Proteins were dialyzed in cassettes [Thermo Scientific Slide-A-Lyser Dialysis Cassettes, 10K molecular weight cut off (MWCO)] overnight at 4°C in 20 mM Tris/HCl (pH 8.0), 150 mM NaCl, 10% glycerol buffer. Proteins were digested immediately to remove the purification tag. Protein concentrations were quantified using the Bicinchoninic acid (BCA) assay (Thermo Scientific Pierce BCA Protein Assay Kit) and a BSA standard curve. One unit of protease per 50 μg fusion protein (Lucigen, SUMO Express Protease) was added and tubes were incubated at 30°C for 1 h. After the cleavage reaction, the SUMO tag and SUMO Express Protease, as well as any residual fusion protein, were removed from the sample by adsorption to a metal affinity chromatography matrix. The sample was applied directly to a Ni-NTA-agarose column to remove His tagged SUMO fragments and the His tagged SUMO protease. The flow-through and wash fractions containing the tag-less PG0720 protein were collected, dialyzed overnight at 4°C in 20 mM Tris/HCl (pH 8.0), 150 mM NaCl, 10% glycerol buffer, and then concentrated with Amicon spin columns (MWCO 10,000). Protein samples were adjusted to 1 mg/ml in 20 mM Tris/HCl (pH 8.0), 150 mM NaCl, 10% glycerol buffer and stored at −20°C.

### Electrophoretic Mobility Shift Assays

EMSAs were performed as described previously [40–42]. Briefly, DNA probes containing the promoter regions of asSuGR or PG0106 were PCR-amplified by primers labeled with biotinylated nucleotides on their 5' end. All PCR products were confirmed by DNA sequencing. For EMSA reactions, each biotin-labeled DNA probe was used with different concentrations of purified PG0720 in a 10-μl reaction mixture containing 10 mM HEPES (pH 7.8), 50 mM KCl, 5 mM MgCl_2_, 1 mM dithiothreitol (DTT), 1 mM EDTA, 1 μg poly(dI-dC), 1 μg bovine serum albumin (BSA), 10% glycerol, and 20 mM acetyl phosphate. The binding reaction was performed at room temperature for 30 min, followed by separation of the DNA-protein samples in a 4.5% non-denaturing, low ionic strength, polyacrylamide gel. The samples were transferred to a Nylon membrane (Thermo Scientific) using a semi-dry transfer apparatus (Bio-Rad). The membrane was air-dried and then exposed to UV light to cross-link biological materials on the membrane. DNA signals were detected using a chemiluminescent nucleic acid detection module kit (Thermo Scientific) as recommended by the supplier.

### DNase I Footprinting Assay

DNase I footprinting assays were carried out using a non-radiochemical capillary electrophoresis method [43]. To find PG0720 binding site(s) in the promoter region of asSuGR, DNA probe containing the promoter region of asSuGR was amplified by primers labeled with 6-FAM (6-carboxyfluorescein) fluorescence on their 5' end and biotinylated nucleotides on their 3' end. The amplified labeled DNA fragment was incubated with PG0720 protein at room temperature for 30 min in a 50-μl reaction mixture identical to that used for EMSAs. DNase I (0.1 unit) digestion was carried out at 37°C for 2 min, and the enzyme reaction was inhibited by adding EDTA to a final concentration of 60 mM, and the samples were heated at 80°C for 10 min. The samples were purified using a MinElute Reaction Cleanup Kit (Qiagen), vacuum dried, resuspended in 10 μl DNase/RNase free water. Fragment analysis was performed by Genewiz, specifically elution samples were subjected to capillary electrophoresis by loading into a 3730xl DNA Analyzer with the LIZ-500 for size standard. Peak Scanner Software v1.0 was used for analyzing electropherograms.

### Invasion Study

Human coronary artery endothelial cells (HCAECs) obtained from Lonza (Walkersville, MD, USA, catalog number CC-2585) were cultured in EGM™-2MV BulletKit™ Medium (Lonza, Walkersville, MD, USA, catalog number CC-3202) and maintained at 37°C/5% CO_2_. Only cells that underwent five or fewer passages were used for experiments. HCAECs were seeded at a concentration of 5 × 10^4^ cells onto 12-well plates containing glass coverslips. After 24 h, the cells were transduced with an adenovirus expression system containing green fluorescent protein (GFP) conjugated to microtubule-associated protein-1 light chain 3B (LC3B) (Welgen, Inc, Worcester, MA, USA) at a multiplicity of infection (MOI) of 200. After 48 h, the cells were either left uninfected or infected at a MOI of 100 with W83, W83*?*PG0106, or W83*?*PG0720. The MOI was confirmed by colony forming unit count on blood agar plates. In order to synchronize the infection, the HCAECs were chilled on ice 15 min prior to inoculation and once infected, the cells were centrifuged at 1,000 *g* at 4°C for 10 min [44]. 1.5 hours post-infection, the cells were treated with 300 μg/mL gentamicin and 400 μg/mL metronidazole in order to remove extracellular bacteria and the antibiotics remained for the duration of the experiment. After 2.5 h post-infection, the HCAECs were fixed with 2% paraformaldehyde dissolved in PBS. The fixed cells were washed three times with PBS mounted on to glass slides with DAPI (Fluoromount-G® Southern Biotech, catalog number 0100-20x). HEAECs were visualized with an Olympus DSU-IX81 Spinning Disc Confocal microscope. The cells were then imaged using QCapture Pro 7 software.

### *Galleria mellonella* Model of Systemic Infection

Larvae of *G. mellonella* (Vanderhorst Wholesale) was used to assess virulence of *P. gingivalis* W83 and its derivatives as described previously [45, 46]. Briefly, groups of 10 larvae (200–300 mg in weight) were injected with 5 μl bacterial inoculum containing ~3 × 10^7^ CFU. After injection, larvae were kept at 37°C and *G. mellonella* survival was recorded at selected intervals for up to 55 h. Experiments were performed independently at least two times with similar results.

## Results

### Response Regulator PG0720 Is Involved in the Presentation of Surface Polysaccharides

**Figure 1:**
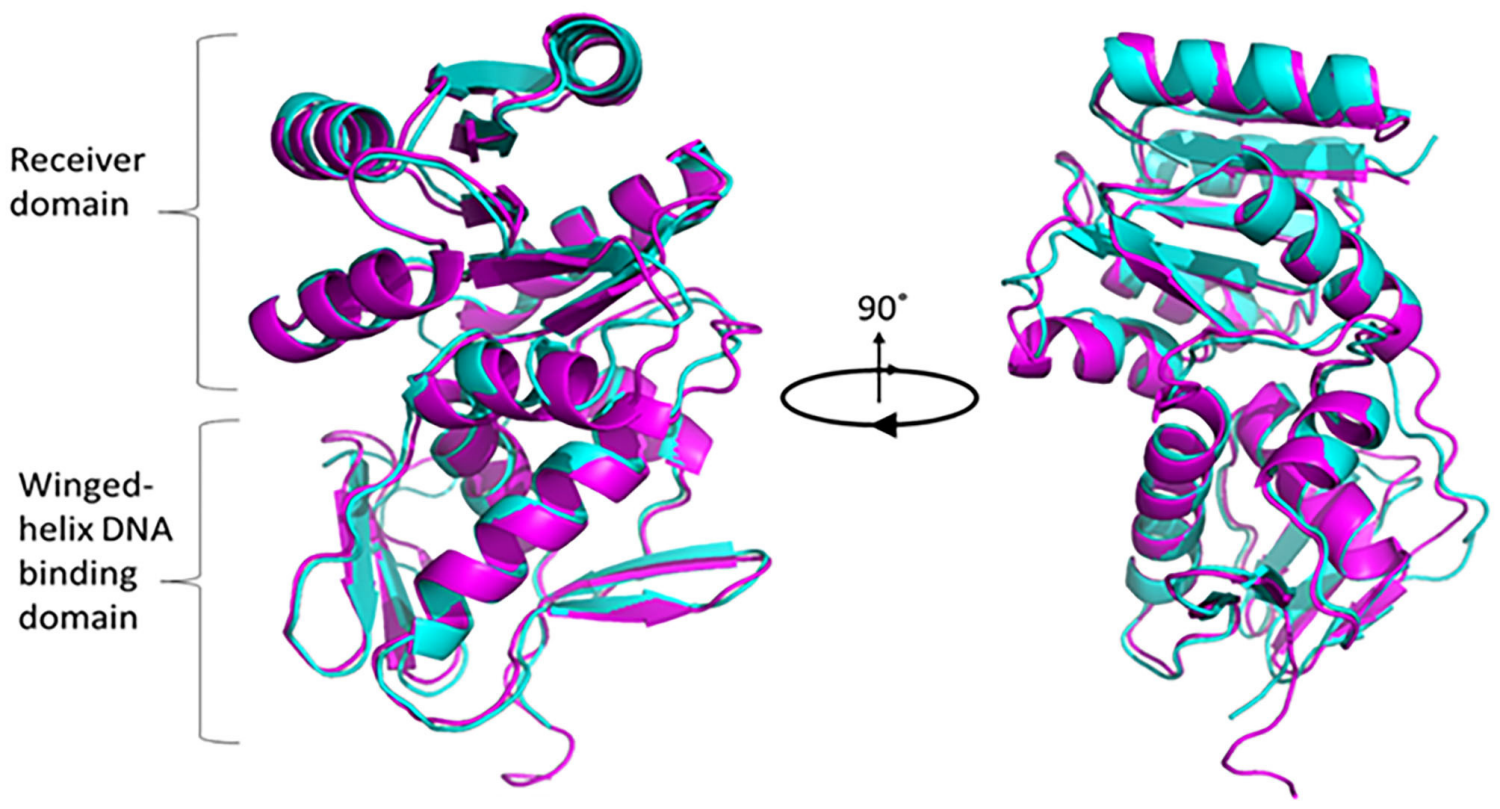
Superimposed modeling for predicted PG0720 structure with crystal structure of PrrA (PDB 1YS6). The TM-scoring of 0.945 in I-TASSER indicates high confidence level of predicted PG0720 to fold as a typical response regulator. PG0720 in magentas while PrrA is in cyan color. Pymol version 2.3.2 was used to render and capture 90° rotational 3D structure.

To understand the structural and functional model for PG0720, we analyzed the protein sequence using alignment and homology searches. Based on the available bioinformatic resources, PG0720 is a typical response regulator consisting of receiver domain at N-terminal end and a winged-helix DNA binding domain at C-terminal end (Supplementary Figure 1A). According to I-TASSER modeling, the predicated structure of PG0720 exhibited the best fit 3D alignment with crystal structure of the response regulatory protein PrrA from *Mycobacterium tuberculosis* (Figure 1, PG0720 in magenta and PrrA in cyan color). The amino acid alignment shows conserved sites in the receiver domain for phosphorylation while variable in DNA binding domain, suggesting DNA sequence specificity. Aspartate residue located between β3 and α3 have been shown to be involved in phosphorylation process (Supplementary Figure 1B) [47].

**Figure 2:**
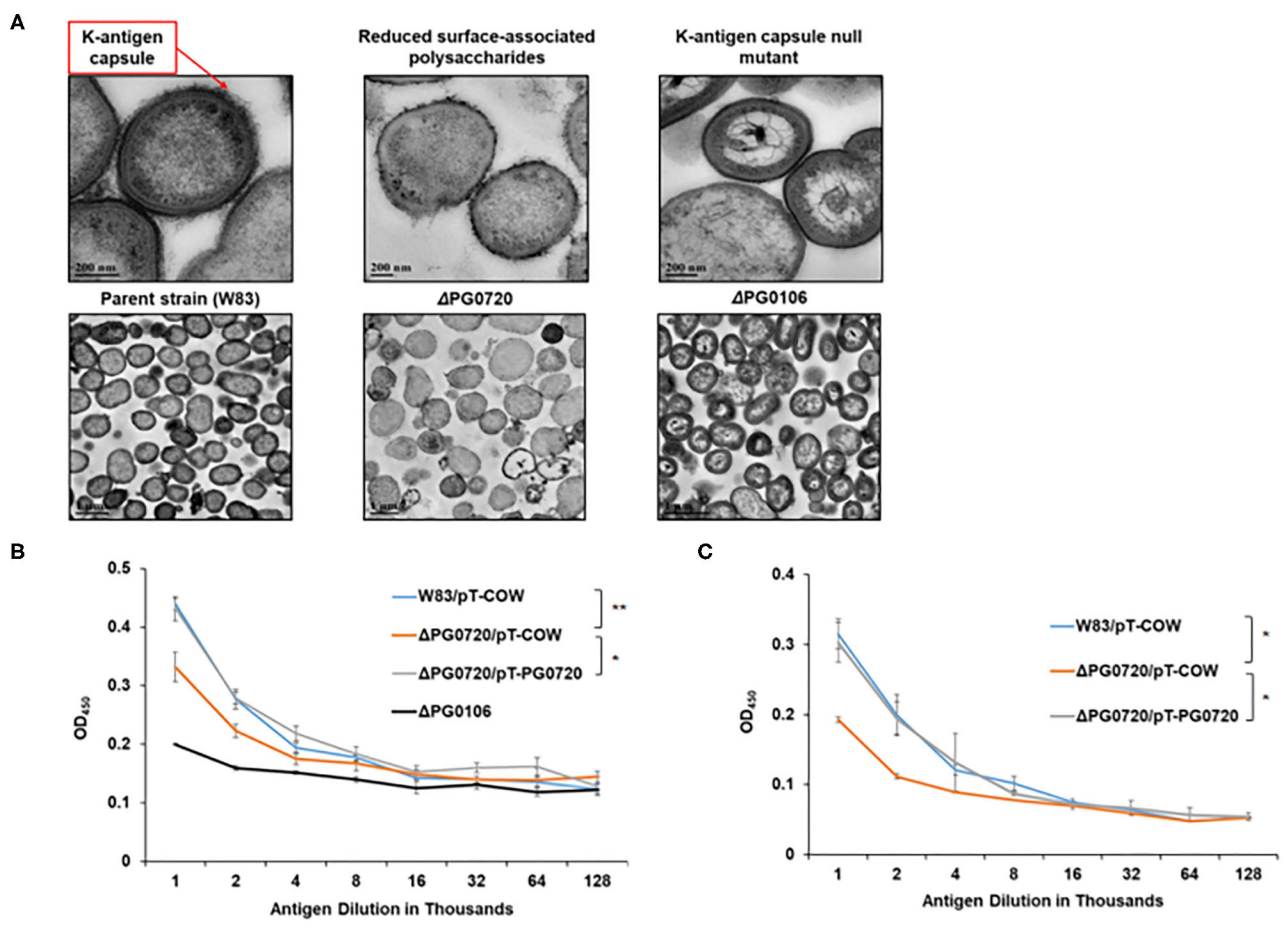
PG0720 mutant produces less surface polysaccharides than the parent strain *P. gingivalis* W83. **(A)** Transmission electron micrographs of the parent strain W83 and the corresponding ΔPG0720, ΔPG0106 mutants. The cells were stained with ruthenium red to detect surface polysaccharides and viewed by transmission electron microscopy. Enzyme-linked immunosorbent assays (ELISAs) were performed to detect K-capsular polysaccharide **(B)** and anionic polysaccharide (A-LPS) **(C)**. Data represent the average of three biological replicates with error bars. The data of the antigen dilution 1/1,000 were analyzed using the Student's *t*-test. *p < 0.05, **p < 0.01.

To evaluate the effect of the loss of PG0720, we generated a W83 PG0720 deletion mutant and compared the mutant with the parent strain and the PG0106 deletion mutant (K-antigen null strain) using transmission electron microscopy. As shown in Figure 2A, ruthenium red staining confirmed an altered expression of surface polysaccharides in the PG0720 mutant strain. In particular, the mutant demonstrated an intermediate amount of capsular polysaccharide, less than the parent strain yet more than the non-encapsulated PG0106 mutant. The growth of the PG0720 mutant strain and the parent strain W83 harboring the empty plasmid (pT-COW) was compared in trypticase soy broth supplemented with hemin and menadione (TSBHK) (Supplementary Figure 2A). A complementation plasmid expressing PG0720 under the control of its native promoter was generated (pT-PG0720) and transferred into the PG0720 mutant. The data show that deletion of PG0720 resulted in a modest reduction in growth rate, and complementation of ΔPG0720 restored the growth defect. To analyze the quantities of surface polysaccharides presented on the surface of the cell by an ELISA format, colonies of the deletion mutant and the parent strain harboring the empty plasmid (pT-COW) or pT-PG0720, and PG0106 mutant were scraped from blood agar plates, normalized by optical density and autoclaved. ELISAs were performed with these preparations using W83 whole-cell polyclonal antiserum to determine the levels of K-antigen capsular polysaccharides (Figure 2B) and 1B5 antiserum to determine the levels of A-lipopolysaccharides (A-LPS) (Figure 2C). As expected, the PG0106 mutant (K-antigen null control) gave very little cross reactivity to the W83 whole-cell polyclonal antiserum. While the PG0720 mutant showed less K-antigen capsular polysaccharides, yet more than the null control. Surprisingly, the PG0720 mutant also showed less cross reactivity with the antibody to A-LPS when compared to the parent strain. The PG0720 complemented strain demonstrated a level of staining comparable to that of the parent strain, showing that deletion of PG0720 is responsible for the K-antigen capsule and A-LPS depletion. Overall, these results support the hypothesis that the surface glycans of *P. gingivalis* are affected by the absence of PG0720.

### Transcription of K-antigen Capsule Synthesis Genes Is Down-Regulated in the Absence of PG0720

**Figure 3:**
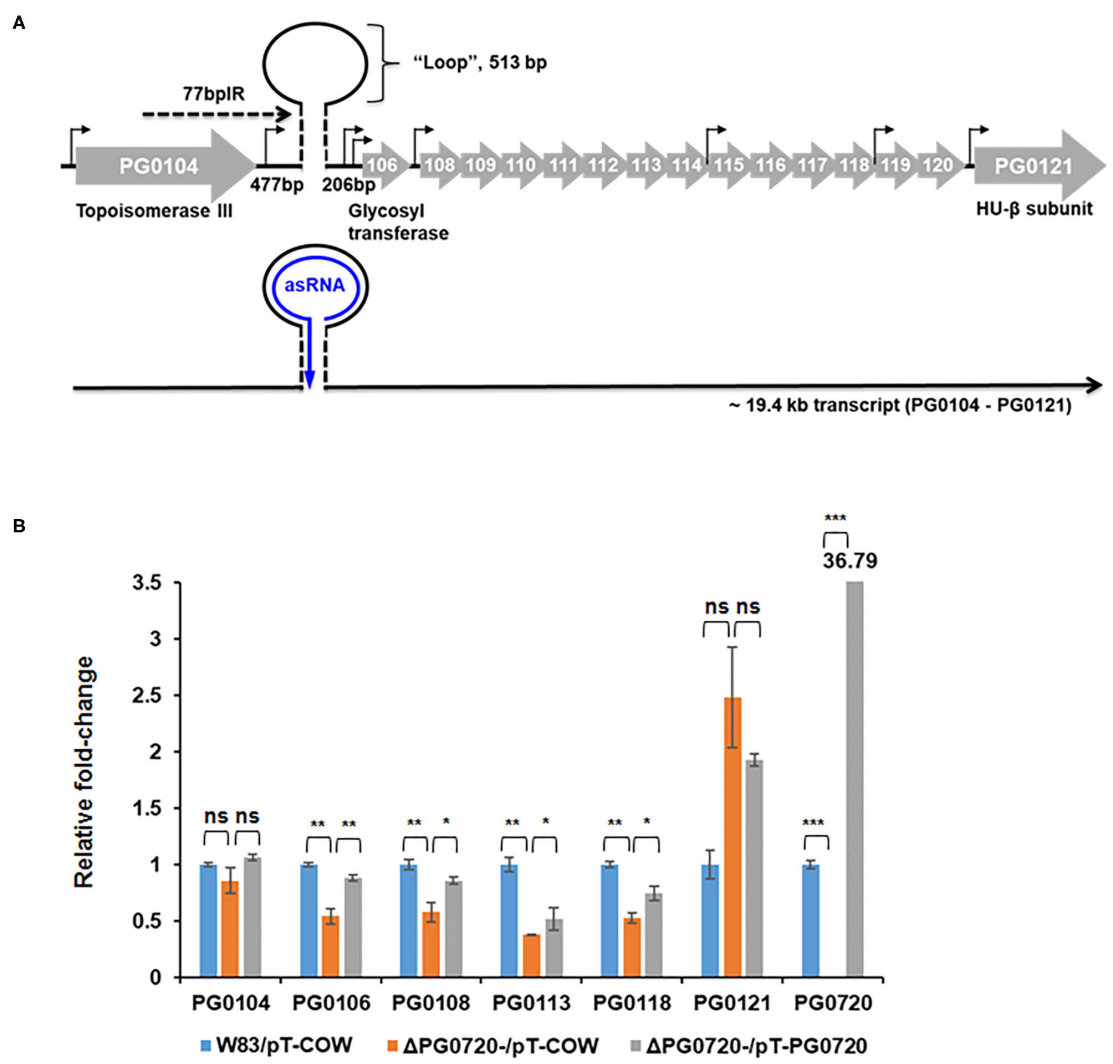
Schematic representation of the K-antigen capsule locus in *P. gingivalis* strain W83 and qPCR of *P. gingivalis* W83, ΔPG0720 containing pT-COW (empty vector control), and complemented ΔPG0720 (pT-PG0720). **(A)** The region of K-antigen capsule locus is predicted to encode 16 open reading frames (PG0104 to PG0121). The position of the predicted hairpin structure created by base pairing of the 77-bp inverted repeat is indicated. The number of base pairs on either side of the hairpin and those contained within the loop are indicated. Promoter regions are identified with black arrows, and the black line at the bottom of the figure represents the large 19.4-kb operon transcript, PG0104-PG0121, which encodes the 77 bpIR element (shown as a stem-loop) near the 5′ end of the transcript. **(B)** The relative transcript levels of K-antigen capsule synthesis genes, including PG0104, PG0106, PG0108, PG0113, PG0118, and PG0121, were determined on the ΔPG0720 deletion mutant harboring the empty plasmid (pT-COW) or pT-PG0720 compared with the transcript levels of the parent strain W83 harboring the empty plasmid (pT-COW). The data represent the mean ± S.D of triplicate determinations. The data were analyzed using the Student's *t*-test. *p < 0.05, **p < 0.01, ***p < 0.001, ns *p* > 0.05.

Since the PG0720 mutant showed less K-antigen capsule and A-LPS, we hypothesized that PG0720 regulates the transcription of the K-antigen capsule locus and the A-LPS locus. To investigate the role of the PG0720 on the transcription of capsule and A-LPS, we performed qPCR analysis. Previously, our research group reported that the 77-bp inverted repeat (77bpIR) region is co-transcribed with the capsule synthesis genes, resulting in a large transcript that is ~19.4 kb (PG0104 to PG0121) (Figure 3A). As shown in Figure 3B, the transcript levels of six of the genes located in this locus (PG0104, PG0106, PG0108, PG0113, PG0118, and PG0121) were examined to evaluate the effect of the PG0720 deletion. The overall expression of genes involved in K-antigen capsule synthesis were down-regulated in the PG0720 mutant compared to those of the parent strain W83. The complemented strain of PG0720 mutant restored the transcript level of those down-regulated genes comparable to those of the parent strain, showing that deletion of PG0720 is involved in regulating the levels of these transcripts.

Because the gene locus involved in the synthesis of A-LPS (PG1135 to PG1142) is highly conserved in various *P. gingivalis* strains [48] with PG1138 being essential for A-LPS biosynthesis, we examined two genes (PG1138 and PG1141) by qPCR analysis (Supplementary Figure 2B). The transcript levels of PG1138 and PG1141 were not significantly altered in the mutant compared to those of the parent strain.

### The Response Regulator PG0720 Binds to the Promoter Region of Antisense RNA in the K-antigen Capsule Locus

**Figure 4:**
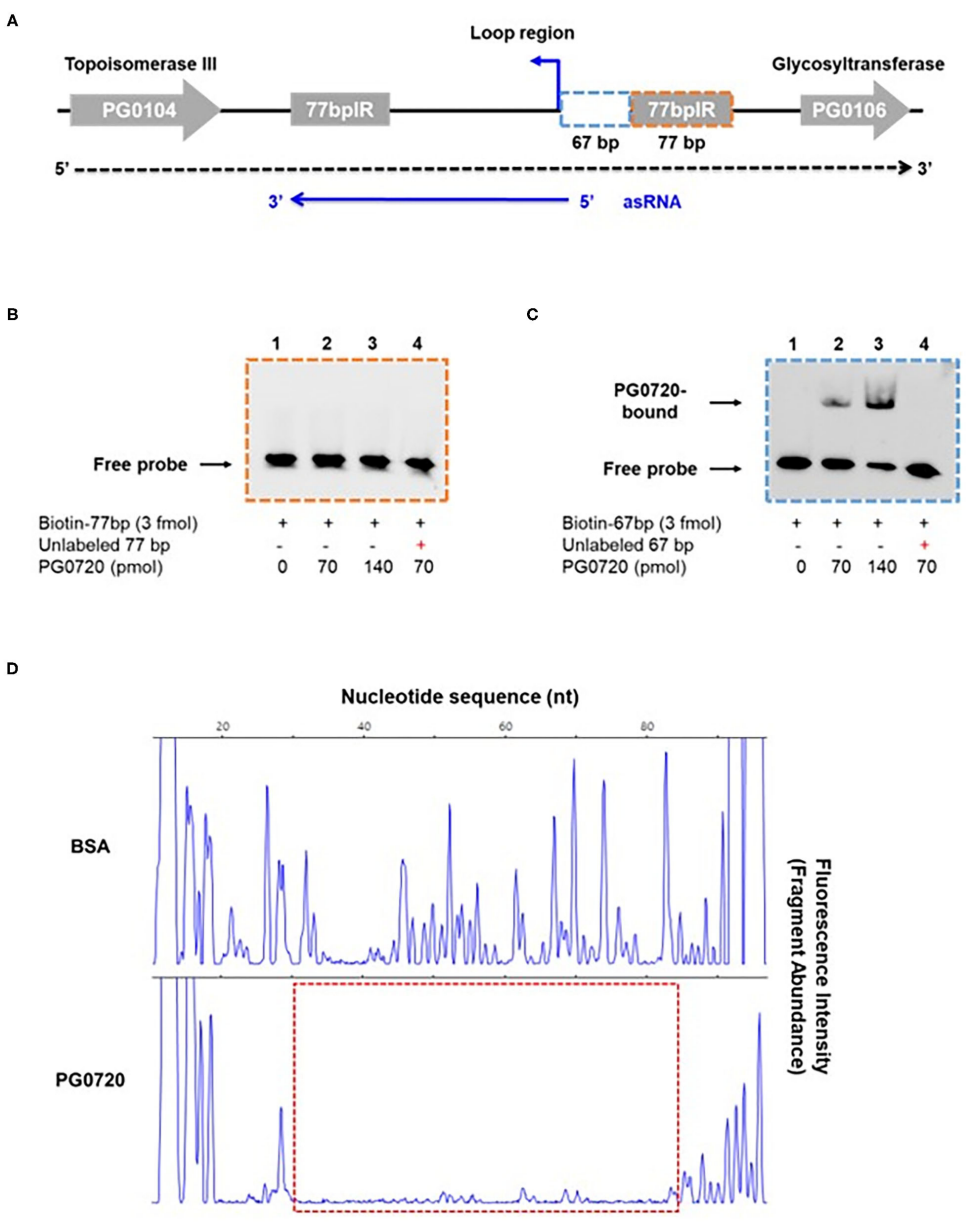
Gel mobility shift analysis and DNase I footprinting assay of PG0720 binding to the predicted promoter region of asSuGR. **(A)** Schematic of the region from PG0104 to PG0106 and the asSuGR transcript. A blue line at the bottom of the figure represents the asSuGR transcript (550 nt). The 5′ end of the asSuGR transcript begins within the loop region (44 bp from the 77 bpIR), and the 3′ end is at the end of the 3′ 77-bp inverted repeat. The predicted promoter regions of asSuGR are outlined with two segmented blue (67-bp) and orange (77-bp) boxes, respectively. **(B,C)** Electrophoretic mobility shift assays (EMSAs) of PG0720 binding with the promoter regions of asSuGR. Biotin-labeled promoter regions (3 fmol) of 77-bp **(B)** or 67-bp **(C)** were incubated with increasing amounts of PG0720 protein (0, 70, and 140 pmol). Unlabelled promoter regions (0.9 pmol) were added to the binding reaction mixtures (lanes 4). The reactions were run on a non-denaturing polyacrylamide gel and the signal observed via chemiluminescence. **(D)** DNase I footprinting assay of PG0720 binding to the promoter region of asSuGR. The electropherograms represent control DNA with BSA (bovine serum albumin) in the upper panels and footprints with of PG0720 in the lower panels. The red segmented box depicts the region protected from DNase I digestion by PG0720.

Since the phosphorylated form of response regulator binds to the promoter of a target gene to either induce or repress gene expression [12, 15], we examined whether phosphorylated PG0720 can directly bind to the promoter region of K-antigen capsule by performing electrophoretic mobility shift assays (EMSAs). In order to test the ability of the protein encoded by PG0720 to bind *in vitro* to the promoter region of K-antigen capsule, a SUMO-tagged recombinant protein was overexpressed and purified. After removing the SUMO-tag, the SDS-PAGE analysis showed successful purification of this protein (~26 kDa) (Supplementary Figure 3A). Mobility shift assays using the purified PG0720 protein were performed as described in Materials and Methods. Two putative promoter sequences of K-antigen capsule (154-bp and 248-bp; in Supplementary Figure 3B) were generated by PCR from strain W83 genomic DNA. Increasing concentrations of phosphorylated PG0720 (0, 35, and 70 pmole) were incubated with promoter sequences (3 fmole). The phosphorylated PG0720 did not bind to the 248-bp promoter region upstream of PG0106 (Supplementary Figure 3C). However, we found that the phosphorylated PG0720 protein bound to the 154-bp promoter region that contains the promoter region of an antisense RNA (asSuGR), causing a shift in mobility on a polyacrylamide gel [Supplementary Figure 3D (lanes 2 and 3)]. The addition of unlabeled probes of the asSuGR promoter to the reaction mixture was able to reduce the presence of the PG0720-shifted 154-bp fragments (Supplementary Figure 3D, lane 4), confirming that the phosphorylated PG0720 binds specifically to the asSuGR promoter region. To identify the precise location of binding site by PG0720 on the 154-bp promoter region, we divided the 154-bp region into the 67-bp and 77-bp region, respectively (Figure 4A). The phosphorylated PG0720 did not bind to the 77-bp region (Figure 4B), while as shown in Figure 4C, the phosphorylated PG0720 protein bound to the 67-bp region, causing a mobility shift on a polyacrylamide gel. The addition of unlabeled probes to the reaction mixture was able to reduce the presence of the PG0720-shifted 67-bp fragments (Figure 4C, lane 4), confirming that the phosphorylated PG0720 binds specifically to the 67-bp promoter region. To delimit the binding site by PG0720 on the asSuGR promoter region, DNase I footprinting experiments using capillary electrophoresis (fragment analysis) were conducted using a PCR-amplified 6-carboxyfluorescein (6-FAM)-labeled DNA probe of the asSuGR promoter region (97 bp) bound to PG0720 under the same conditions as used for EMSAs (Figure 4D). Sites protected from DNase I digestion by PG0720 were visualized as regions lacking discernable peaks compared to a control reaction mixture containing BSA (bovine serum albumin). Reaction mixtures containing PG0720 yielded only a single region of protection spanning from −365-bp to −314-bp, with respect to the ATG start site of the PG0106 gene. This protected region largely corresponds to the predicted 67-bp asSuGR promoter region from the EMSA (Figure 4C), confirming its involvement in binding with PG0720.

### PG0720 Upregulates the Expression of asSuGR Which Corresponds With an Increase in the Expression Level of Genes in the K-antigen Capsule Locus

**Figure 5:**
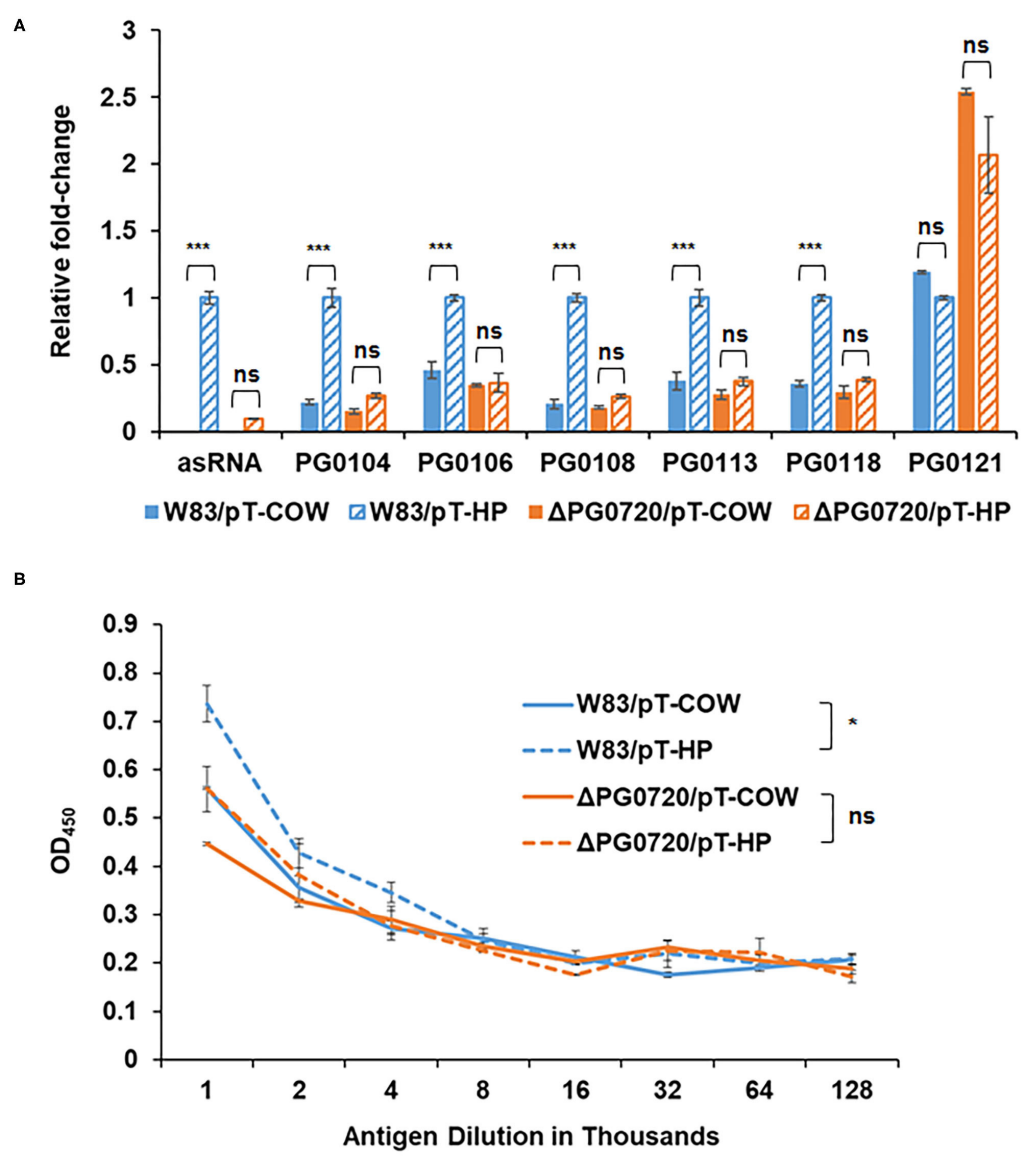
qPCR to validate the effects of asSuGR overexpression on the expression levels of K-antigen capsule synthesis genes and ELISA showing increased synthesis of K-antigen capsule when asSuGR is overexpressed. **(A)** The relative transcript levels of asSuGR and K-antigen capsule synthesis genes, including PG0104, PG0106, PG0108, PG0113, PG0118, and PG0121, were determined on the strain W83 and ΔPG0720 mutant harboring the empty plasmid (pT-COW) or pT-HP overexpresses the asSuGR. The results are presented as the relative levels (mean ± S.D. of triplicate determinations) compared with the transcript levels of the parent strain W83 harboring the plasmid pT-HP. The data were analyzed using the Student's *t*-test. ***p < 0.001, ns *p* > 0.05. **(B)** K-antigen capsule content of strain W83 and ΔPG0720 mutant with plasmid pT-COW or pT-HP was quantitated by ELISA. The strain overexpressing the asSuGR has increased amounts of K-antigen capsule compared to the control strain. The data represent the mean ± S.D of triplicate determinations. The data of the antigen dilution 1/1,000 were analyzed using the Student's *t*-test. *p < 0.05, ns *p* > 0.05.

As noted, an asSuGR encoded between the 77-bp inverted repeats was previously identified [26]. As shown in Figure 4A, this RNA molecule is 550 nucleotides in length and has an internal 32-bp inverted repeat. To explore the function of the asSuGR between the 77-bp inverted repeats, we generated a *P. gingivalis* pT-COW plasmid harboring the DNA (1,408 bp) between PG0104 and PG1016. This plasmid, designated pT-HP was transformed into the ΔPG0720 mutant strain as well as the parent strain W83 (to generate an asSuGR-overexpressing strain). As shown in Figure 5A, strain W83 harboring pT-HP overexpresses the asSuGR, and the strain showed enhanced expression levels of the K-antigen capsule genes compared to the control parent strain harboring plasmid pT-COW, indicating that the asSuGR is a *trans*-acting molecule. In addition, overexpression of the asSuGR also resulted in increased reactivity to K-antigen capsule (Figure 5B). Importantly, the ΔPG0720 mutant harboring pT-HP expressed less asSuGR compared to the parent strain harboring pT-HP. In essence, the mutant strain showed similarly low levels of expression of K-antigen capsule genes regardless of the presence of pT-HP. Overall, these results support the hypothesis that the expression level of asSuGR between PG0104 and PG0106 of *P. gingivalis* can be activated by the presence of PG0720 and the enhanced expression level of asSuGR results in higher transcript levels of K-antigen capsule genes.

### PG0720 Regulates Virulence and Intracellular Trafficking

**Figure 6:**
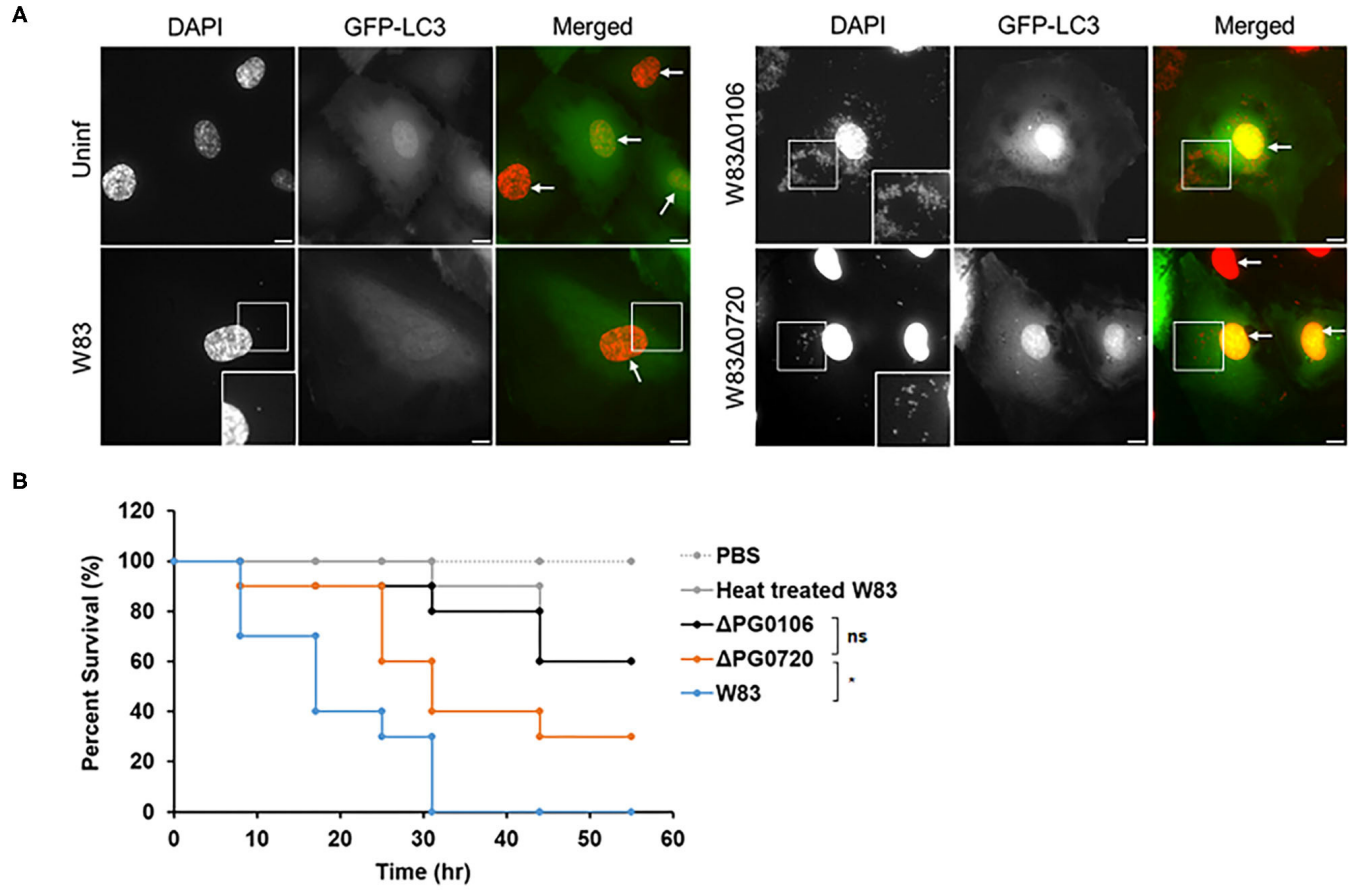
Intracellular trafficking of *P. gingivalis* in HCAEC and survival rates of *Galleria mellonella* larvae injected with *P. gingivalis* parent strain W83 or its derivatives. **(A)** Representative microscopic images of *P. gingivalis* strain W83, PG0106, and PG0720 (red dots) with autophagosome specific GFP-LC3 (green) at 2.5 h post-inoculation. *P. gingivalis* cells (white boxes) and nuclei of HCAECs (white arrows) were stained with DAPI (pseudocolored red). The white boxes at the bottom of the DAPI panels are magnified versions of the smaller white boxes in the middle of the DAPI panels. Scale bar is equivalent to 10 μm. **(B)** Larvae injected with PBS or heat treated W83 were used as negative controls. Larvae infected with ΔPG0720 mutant showed slightly reduced survival rate compared with the parent strain (*n* = 10 larvae per treatment). The average of three replicates is shown. The data were analyzed using the Student's *t*-test. *p < 0.05, ns *p* > 0.05.

In the previous study [49], it was shown that *P. gingivalis* strain 381 (non-encapsulated strain) activates autophagy in human coronary artery endothelial cells (HCAECs) whereby the autophagosome provides a replicative niche for *P. gingivalis* within these host cells during invasion [49]. Studies from the laboratory of Dr. Ann Progulske-Fox (APF) have shown that *P. gingivalis* strain W83 primarily migrates through the autophagic pathway during the invasion of HCAEC [50, 51]. However, when we compared the expression level of K-antigen capsule between two *P. gingivalis* W83 strains, one from the APF laboratory (from SUNY-Buffalo collection, Buffalo, NY) and the other from Dr. Mary Ellen Davey's (MED) lab collection (Christian Mouton, Laval University, Quebec City, Quebec, Canada), only the MED strain produced K-antigen capsule, the APF W83 strain was K-antigen null, like the ΔPG0106 mutant (Supplementary Figure 4A). To determine if the production of K-antigen capsule or the deletion of PG0720 altered the intracellular trafficking of W83, the internalized bacterial cells within autophagosomes of HCAEC were evaluated (Figure 6A). The microtubule-associated protein1 light chain 3 (LC3) was used as a specific marker for autophagosomes [51, 52]. The majority of ΔPG0106 cells were found within LC3 positive vacuoles and as expected, the W83 cells from the APF lab (non-capsulated) were also found within LC3 positive vacuoles (Supplementary Figure 4B). In contrast, there were dramatically fewer cells of the MED W83 parent strain found within HCAE cells when compared to the ΔPG0720 mutant or the ΔPG0106 mutant. The PG0720 mutant showed an intermediate number of bacterial cells in LC3 positive vacuoles. These data indicate that the W83 cells can traffic through the autophagic pathway during the invasion of HCAEC, but only when the K-antigen capsule is down-regulated or absent; suggesting that the cell surface presentation of K-antigen is down-regulated during the process of HCAEC invasion and under routine laboratory conditions, *P. gingivalis* strains that synthesize K-antigen capsule are defective in invasion of HCAECs.

### Production of Surface Glycans Impacts the Virulence of *P. gingivalis* in *Galleria mellonella* Greater Wax Moth Larvae Model

To investigate the significance of surface glycans regulated by PG0720 in *P. gingivalis* virulence, we used the *Galleria mellonella* larvae model that possesses an innate immune system. As shown in Figure 6B, virulence of the PG0720 mutant was reduced with ~30% larvae survival compared to the parent strain after 55 h of infection. The ΔPG0106 capsule null mutant killed *G. mellonella* at rates comparable to the heat-treated parent strain that was used as a negative control for virulence, with similar averages of larvae survival (~60%) after 55 h of infection. Consequently, these findings indicate that the level of expression of K-antigen capsule increases the virulence of *P. gingivalis* strain W83.

### PG0720 Has a Global Impact on Heme Acquisition, Transcriptional Regulation, Signaling, and Metabolism

To identify differentially expressed genes linked to PG0720, the gene expression profiles for the parent strain W83 and the ΔPG0720 mutant were determined by RNA-seq analysis of cells grown as colony biofilms. The data showed that the overall expression of genes involved in K-antigen capsule synthesis were down-regulated in the PG0720 mutant compared to those of the parent strain W83, however the *p*-values were higher than 0.05; therefore expression of these genes was further analyzed by qPCR; Figure 3B. As shown in Table 1, a total 28 genes were found to be differentially expressed more than 1.5-fold (*p* < 0.05) in the ΔPG0720 mutant compared with the parent strain when grown on a TSBHK blood agar surface. Among these genes, 6 genes were up-regulated and 22 genes were down-regulated (Supplementary Figure 5). Importantly, the expression of a number of genes involved in iron acquisition was down-regulated in the ΔPG0720 mutant compared with the parent strain, specifically HmuY (PG1551), which is a predicted heme-binding protein. This result is consistent with a previous study [15], which showed that the regulon of this two-component system contained genes that were involved in hemin acquisition in *P. gingivalis* strain ATCC 33277. In addition, a few genes involved in controlling transcription (PG0214, PG1007, and PG1622) were down-regulated in the ΔPG0720 mutant. Notably, the mutant showed significant down-regulation (log2 fold change −4.25) of PG1007 which is a predicted GntR family transcriptional regulator and this result is also consistent with the previous study [15], which showed that the two-component system regulates the expression of PGN_1346 (PG1007). Lastly, although the *p*-values were higher than 0.05, the RNA-seq data showed that genes flanking other copies of the 77 bpIR element on the chromosome (PG0498 and PG0880) were down-regulated in the ΔPG0720 mutant, which is also consistent with the previous study [15]. Interestingly, further analysis using qPCR of PG0498 and PG0880 in cells grown to late exponential phase showed that PG0498 (*luxS*) was up-regulated in the parent strain harboring pT-HP (over-expression of the asRNA), while PG0880 was unaffected (Supplementary Figure 6). Although further studies are required to elucidate the role of these 77 bpIR elements, the data suggests that the asSuGR molecule influences the transcript levels of genes involved in both glycan synthesis and AI-2/quorum sensing. In contrast, a few genes involved in signaling and metabolism, including PG0707, PG0719, PG0143, PG0497, PG0629, and PG1310 were up-regulated in the ΔPG0720 mutant. Altogether, expression of genes involved in heme acquisition, transcriptional regulation, signaling and metabolism showed significant changes in the ΔPG0720 mutant compared with the parent strain. These results support the hypothesis that the differentially regulated genes in the ΔPG0720 mutant were potential components of PG0720 regulon.

**Table 1:** RNA-seq analysis and differential gene expression of ΔPG0720 mutant vs. *P. gingivalis* parent strain W83 (*p* < 0.05).

**Annotation**	**Common name**	**Predicted product**	**Log2 fold change^a^ (ΔPG0720/WT)**
**Target gene for deletion**			
PG0720		Response regulator transcription factor	−3.00
**Transcription/translation**			
PG0214		sigma-70 family RNA polymerase sigma factor	−0.68
PG1007	–	GntR family transcriptional regulator	−4.25
PG1622		DNA gyrase/topoisomerase IV subunit A	−0.80
**Iron/virulence**			
PG1151	–	Iron-containing alcohol dehydrogenase	−0.71
PG1551	–	Heme-binding protein HmuY	−0.83
**Transport/signaling**			
PG0707	–	TonB-dependent receptor	0.59
PG0719	–	HAMP domain-containing histidine kinase	1.15
PG1798	–	T9SS type A sorting domain-containing protein	−0.65
**Enzyme/metabolism**			
PG0135	rsmA	16S rRNA [adenine(1518)-N(6)/adenine(1519)-N(6)]-dimethyltransferase RsmA	−1.01
PG0143	–	Carbon-nitrogen hydrolase	0.87
PG0343	megL	Methionine gamma-lyase	−0.59
PG0445	pepT	Peptidase T	−0.75
PG0497	–	5'-methylthioadenosine/adenosylhomocysteine nucleosidase	0.82
PG0583	–	Cell division protein FtsA	−0.59
PG0629	–	NAD kinase	0.81
PG1310	queC	7-cyano-7-deazaguanine synthase QueC	0.74
PG1435	–	Site-specific integrase	−0.64
PG2014	cas1b	Type I-B CRISPR-associated endonuclease Cas1	−3.79
PG2069	–	SDR family oxidoreductase	−0.59
**Others**			
PG0217	–	Hypothetical protein	−1.20
PG0218	–	Hypothetical protein	−0.86
PG0987	–	DUF4252 domain-containing protein	−1.04
PG1492	–	GLPGLI family protein	−1.14
PG1493	–	Hypothetical protein	−0.81
PG1508	–	Hypothetical protein	−0.75
PG1582	–	VWA domain-containing protein	−0.65
PG1908	–	GLPGLI family protein	−1.18

a *Difference of > 1.5-fold*.

## Discussion

**Figure 7:**
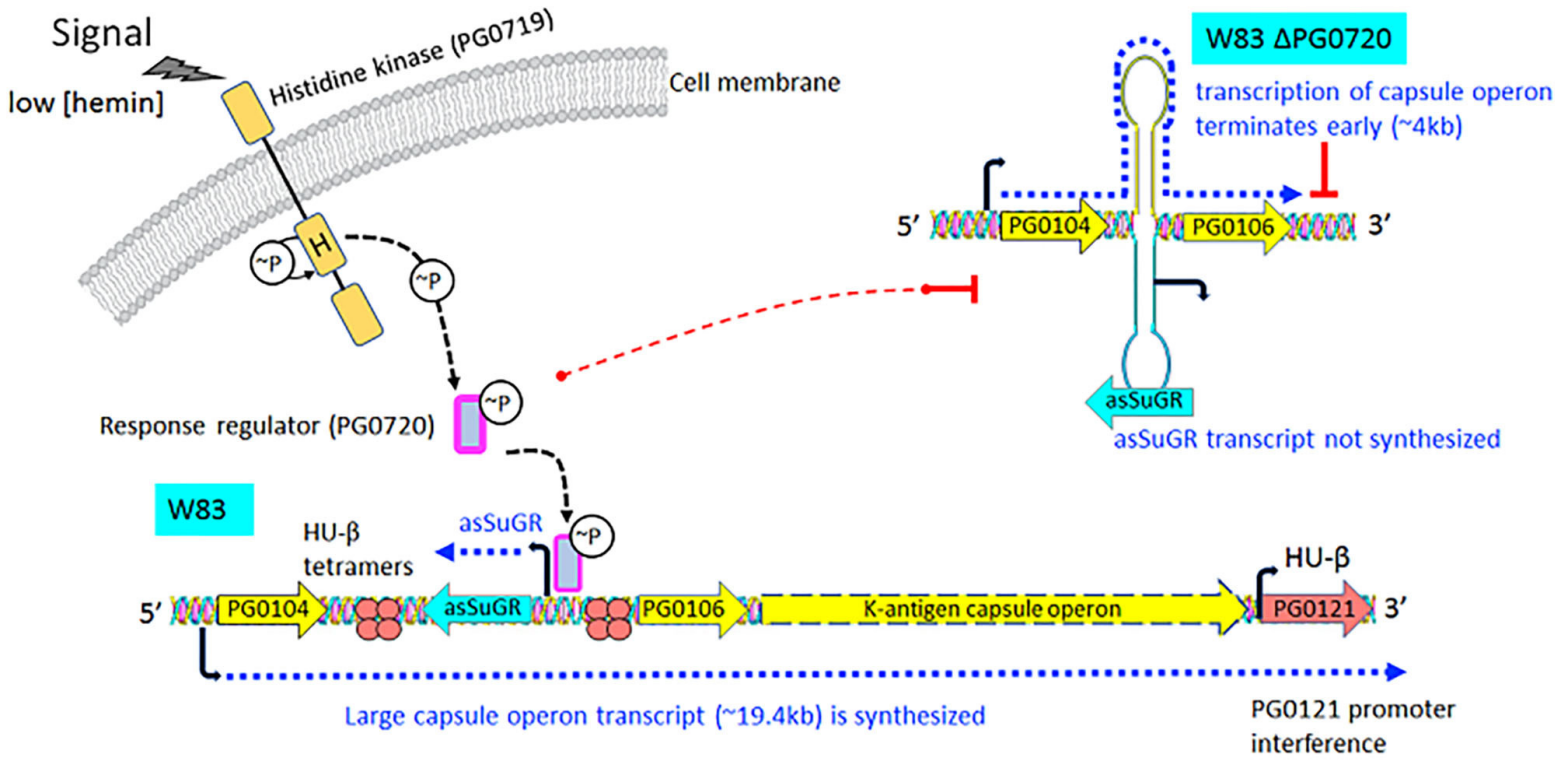
Proposed working model describing the impact of transcription of the antisense molecule asSuGR on transcription of the K-antigen capsule operon on the sense strand. When the two-component sensor kinase (PG0719) senses low levels of hemin, it autophosphorylates and then transfers the phosphate to its cognate response regulator PG0720. Phosphorylated PG0720 activates transcription of asSuGR. When asSuGR is transcribed (W83 parent strain), the large capsule operon transcript (~19.4 kb) on the sense strand is synthesized and this in turn interferes with activation of the PG0121 promoter. When asSuGR is not transcribed (W83 ΔPG0720), synthesis of the large capsule operon is terminated earlier. The working hypothesis is that processivity i.e., the uninterrupted transcription by RNA polymerase, is a challenge for transcription of the large capsule operon transcript on the sense strand. Hence, transcription and processivity are regulated via a mechanism of antitermination. The data indicate that asSuGR along with PG0121 (DNABII- DNA binding and bending protein; HU-β) support processivity and synthesis of this large transcript. In addition, since asSuGR works *in trans*, the antisense may also bind to the sense strand after it is transcribed, thereby influencing stability.

Transcriptomic studies have shown that bacteria synthesize an array of antisense regulatory RNAs [53]. These molecules vary greatly in their size, their location in respect to sense strand genes, and the mechanisms by which they impact gene expression levels [53]. A variety of bacterial antisense RNAs have been characterized and shown to play a role in regulating motility, iron acquisition, and biofilm development [54]. In this study, we determined that a two-component response regulator (PG0720) directly binds the promoter region and activates expression of an antisense transcript (asSuGR) located within an unusual 77 bpIR element. Expression of asSuGR correlates with an increase in the transcript levels of genes on the sense strand (genes encoding proteins required for synthesis of K-antigen capsule), suggesting that asSuGR may stabilize the sense transcript(s) [54–57]. Importantly, the sense-strand capsule operon transcript is very large (~19.4 kb), so processivity i.e., the uninterrupted transcription of this mRNA molecule by RNA polymerase, is a challenge. Our working hypothesis has been that synthesis is controlled by an anti-termination mechanism and recruitment of elongation factors. The data presented in this report provide further support of this hypothesis. Based on our earlier studies, including Northern analysis of transcripts encoded in this locus [24–26, 58, 59], our current working model (Figure 7) is that phosphorylated PG0720 activates transcription of asSuGR under condition of oxidative stress or low hemin. And when asSuGR is transcribed, the large capsule operon transcript on the opposing sense strand is synthesized, which also interferes with activation of the downstream PG0121 promoter, resulting in low expression levels of PG0121. When asSuGR is not transcribed (W83ΔPG0720) synthesis of the large capsule operon is terminated earlier (~4 kb transcript) and expression from the PG0121 promoter at the 3'-end of the locus is up-regulated (shown in Figures 3, 5A). Currently, our working model is that asSuGR along with PG0121 (DNABII- DNA binding and bending protein; HU-β) work in concert as part of an antitermination mechanism. Experiments to identify other proteins involved in this regulatory mechanism are on-going.

Interestingly, not only the amount of K-antigen capsule but also the level of A-LPS was reduced by the deletion of PG0720. These results align with our previous study showing that deletion of the 77 bpIR element that encodes asSuGR resulted in lower levels of K-antigen capsule and A-LPS [26]. Importantly, asSuGR can function *in trans* when expressed from a plasmid and there are multiple copies of the 77 bpIR element on the chromosome [26]. To explore whether or not the regulation is at the level of transcription, expression of two genes (PG1138 and PG1141) involved in A-LPS synthesis [60] were examined in the PG0720 deletion mutant compared to the parent strain. Expression of these genes was found to be unchanged, so it is not yet clear how asSuGR impacts the levels of A-LPS. It should be noted, however, that one of the potential asSuGR targets is located in proximity to PG1780, a gene that encodes a serine palmitoyl transferase (SPT) which is required for synthesis of *P. gingivalis* sphingolipids. Findings from an earlier study showed that the sphingolipid null PG1780 mutant produced low levels of K-antigen capsule, yet more A-LPS when compared to the parent strain W83 [27]. The proximity of SPT to a predicted asSuGR target as well as the altered surface polysaccharides of the ΔPG1780 mutant make it tempting to speculate that synthesis of sphingolipids may be coordinately regulated and play a role in the deployment and/or anchoring of K-antigen capsule to the outer membrane and this in turn can indirectly affect the levels of A-LPS. Overall, the data indicate a strong link between the levels of surface glycans and transcription of asSuGR by PG0720 and that the RNA molecule asSuGR regulates the levels of capsule by directly controlling the transcript levels of genes involved in synthesis and indirectly by regulating the membrane lipid composition.

Bacterial surface glycans play a central role in immune modulation and evasion [61]. Our results show that a *P. gingivalis* mutant that is defective in iron acquisition and the presentation of surface glycans is more invasive (endothelial cells), yet less virulent when tested in the *Galleria mellonella* infection model. Studies have shown that A-LPS mutants are more susceptible to killing by the host complement system [62, 63]; suggesting that the change in virulence we observed in the *G. mellonella* model may be due to a lack of A-LPS. In addition, although both the O-LPS and A-LPS induce a host immune response, the response to lipid A of total LPS is significantly stronger than that of A-LPS alone [17]. Hence the low levels of A-LPS produced by PG720 mutant may result in a strain that is more readily recognized and cleared by the host. In contrast, however, K-antigen capsule does not provide protection to killing by the complement system [62], yet K-antigen encapsulated strains are described as more virulent. Encapsulated strains disseminate causing a spreading type of infection in mice [64, 65]. In general, capsules can either facilitate or prevent bacterial adherence to abiotic and biotic surfaces. Most *P. gingivalis* invasion studies have used strain 33,277, which is non-encapsulated. Here, we discovered that although earlier studies showed that strain W83 is able to invade endothelial cells, only W83 isolates that are not able to produce a capsule can do so. This finding aligns with previous reports showing that the encapsulated strain W83 is a poor colonizer of abiotic or biotic surfaces [58, 66–68].

The previous study on this two-component system using strain 33,277 showed that PGN_0753 (PG0720) controls a suite of genes involved in acquisition and uptake of iron/hemin and the system was designated HaeSR (for haemin) [15]. The critical role of iron in the pathogenesis of *P. gingivalis* has been extensively studied [69–71]. This anaerobe uses hemoglobin as a source of iron more effectively than other iron sources [72], and has a number of mechanisms for sequestering heme from hemoglobin and other host proteins via degradation and heme binding by gingipains, and outer membrane receptors with high affinity for heme [69]. As shown in Table 1, we demonstrated that PG0720 regulon in strainW83 also involved acquisition and transport of iron/hemin into cells. These results support our working hypothesis that PG0720 can regulate not only hemin acquisition but also the synthesis of surface glycans in *P. gingivalis* strain W83. Future studies of the PG0717-PG0720 locus have the potential to disclose how complex environmental signals are integrated into the regulatory networks modulating *P. gingivalis* virulence and homeostasis at the cellular and/or community level.

## Data Availability Statement

The datasets presented in this study can be found in online repositories. The names of the repository/repositories and accession number(s) can be found in the article/Supplementary Material.

## Author Contributions

H-MK contributed to the conception, experimental design, data acquisition, interpretation of data, and manuscript preparation. DR contributed to the experimental design, data acquisition, interpretation of data, and editing of the manuscript. AW and HG contributed to the data acquisition, interpretation of data, and editing of the manuscript. AP-F contributed to the interpretation of data and editing of the manuscript. MD contributed to the conception, experimental design, interpretation of data, and editing of the manuscript. All authors contributed to the article and approved the submitted version.

## Conflict of Interest

The authors declare that the research was conducted in the absence of any commercial or financial relationships that could be construed as a potential conflict of interest.
