# Supplementary material for: A Novel Regulation of K-antigen Capsule Synthesis in Porphyromonas gingivalis Is Driven by the Response Regulator PG0720-Directed Antisense RNA
Source: Front Oral Health. 2021 Jul 1;2:701659. doi: 10.3389/froh.2021.701659 (PMC8757827; doi:10.3389/froh.2021.701659)
Supplement: Supplementary file 1 [file Data_Sheet_1.docx]

**
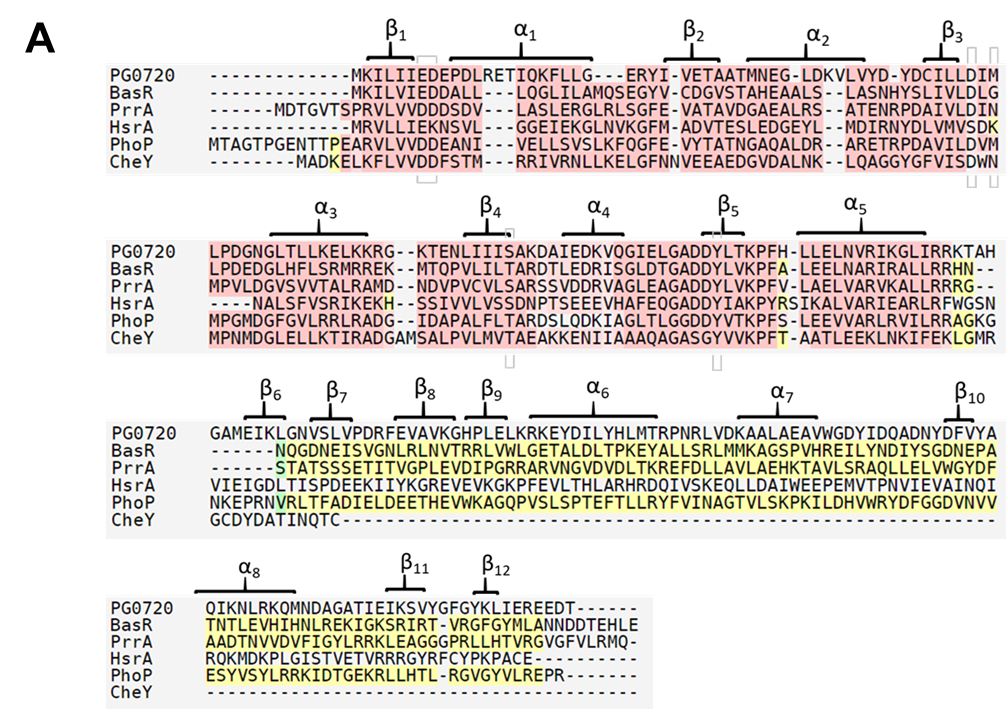
**

**
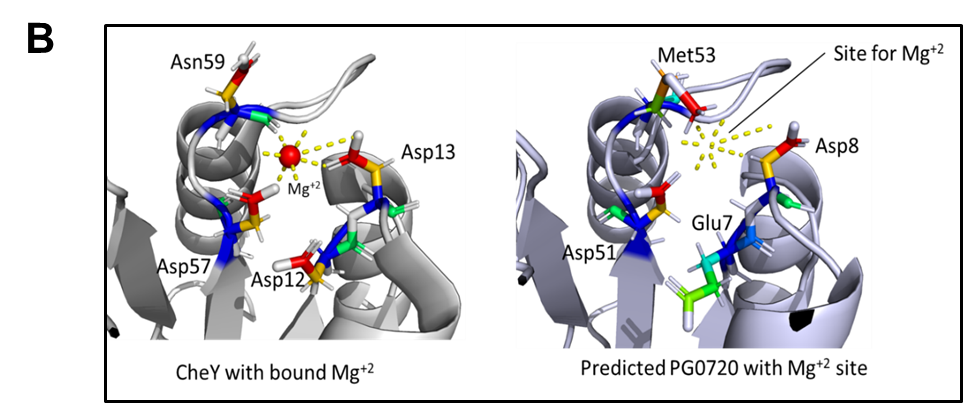
**

**Supplementary Figure 1.** Computational modeling of PG0720 for identifying phosphorylation and DNA binding sites. **(A)** Alignment of response regulators with known crystal structures identified by I-TASSER. The sequences were obtained by NCBI database and software Expresso (Tcoffee) was used to align them. **(B)** Comparison of Mg+2 bound site between CheY (PDB 6TGY) and predicted structure of PG0720. Pymol version 2.3.2 was used to render and compare the structure and residues between CheY and PG0720.


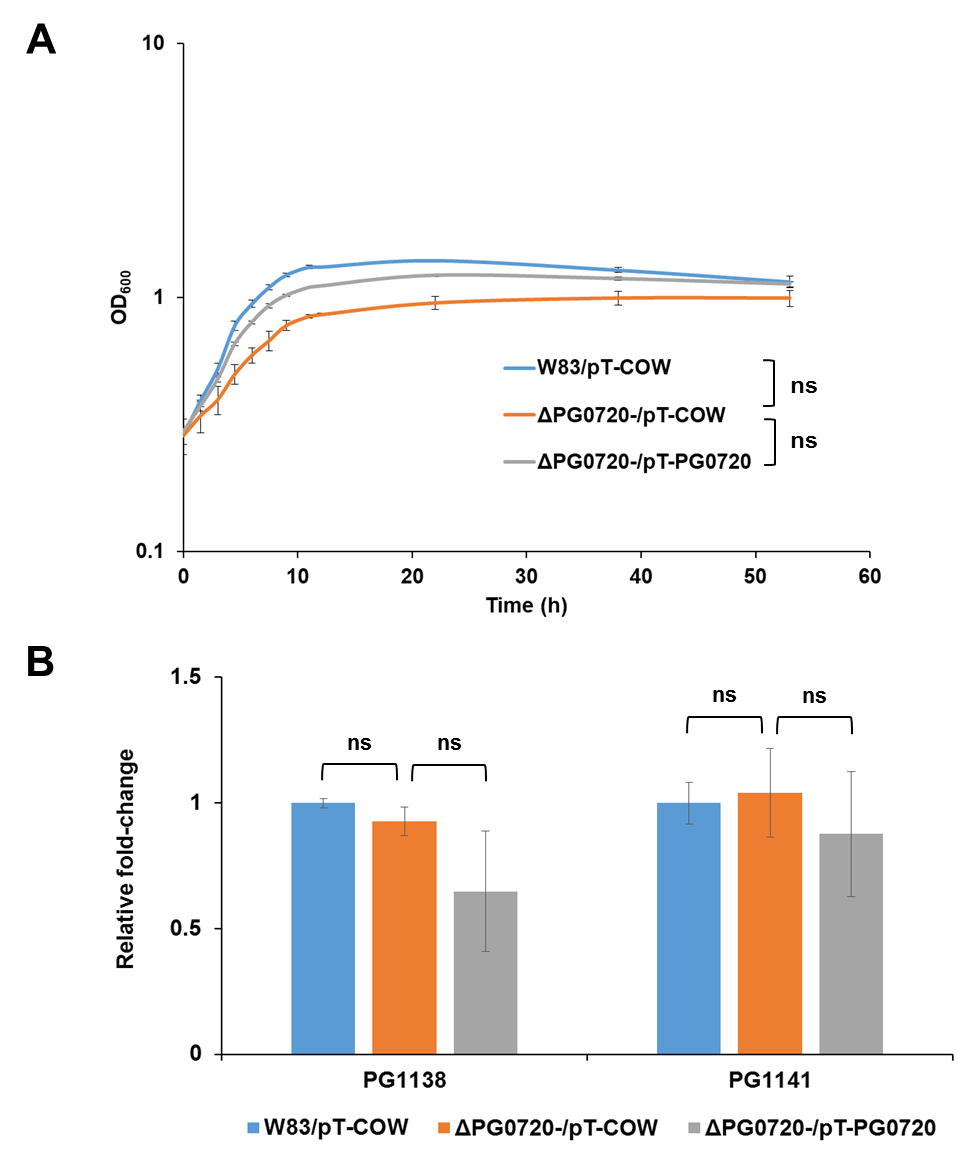


**Supplementary Figure 2.** Assessment of growth phenotypes and quantitative PCR (qPCR) of *P. gingivalis* W83, ΔPG0720 containing pT-COW (empty vector control), and complemented ΔPG0720 (pT-PG0720). The data were analyzed using the Student’s *t* test. ns *p* > 0.05. **(A)** *P. gingivalis* W83/pT-COW (blue), ΔPG0720/pT-COW (orange), ΔPG0720/pT-PG0720 (black) were grown in TSBHK media in the presence of tetracycline. The data represent the mean ± S.D of triplicate determinations. **(B)** Both PG1138 and PG1142, located in the PorR locus, have been reported to affect the presence of A-LPS. The relative transcript levels of genes, including PG1138 and PG1142, were determined on the ΔPG0720 deletion mutant harboring the empty plasmid (pT-COW) or pT-PG0720. The results are presented as the relative levels (mean ± S.D. of triplicate determinations) compared with the transcript levels of the parent strain W83 harboring the empty plasmid (pT-COW).


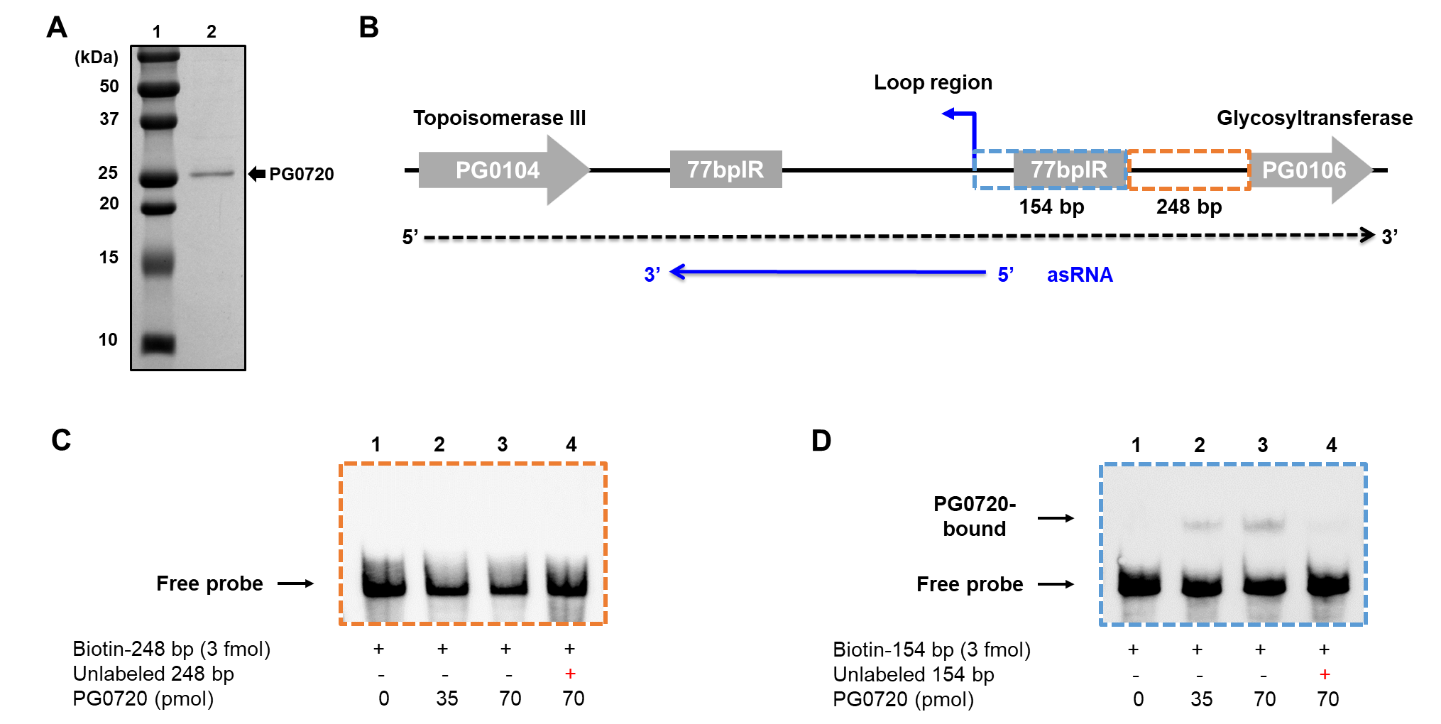


**Supplementary Figure 3.** Purification of the PG0720 protein (26.2 kDa) and gel mobility shift analysis of PG0720 binding to the predicted promoter regions. **(A)** SDS-PAGE analysis results, showing purification of the PG0720 protein. **(B)** Schematic of the region from PG0104 to PG0106 and the asSuGR transcript. A blue dashed line at the bottom of the figure represents the asSuGR transcript (550 nt). The 5′ end of the asSuGR transcript begins within the loop region (44 bp from the 77bpIR), and the 3′ end is at the end of the 3′ 77-bp inverted repeat. The predicted promoter regions of asSuGR and PG0106 are outlined with two segmented blue (154-bp) and orange (248-bp) boxes, respectively. **(C-D)** Electrophoretic mobility shift assays (EMSAs) of PG0720 binding with the promoter regions of asSuGR and PG0106. Biotin-labelled promoter regions (3 fmol) of 248-bp **(C)** or 154-bp **(D)** were incubated with increasing amounts of PG0720 protein (0, 35, and 70 pmol). Unlabelled promoter regions (0.9 pmol) were added to the binding reaction mixtures (lanes 4). The reactions were run on a non-denaturing polyacrylamide gel and the signal observed via chemiluminescence.

**
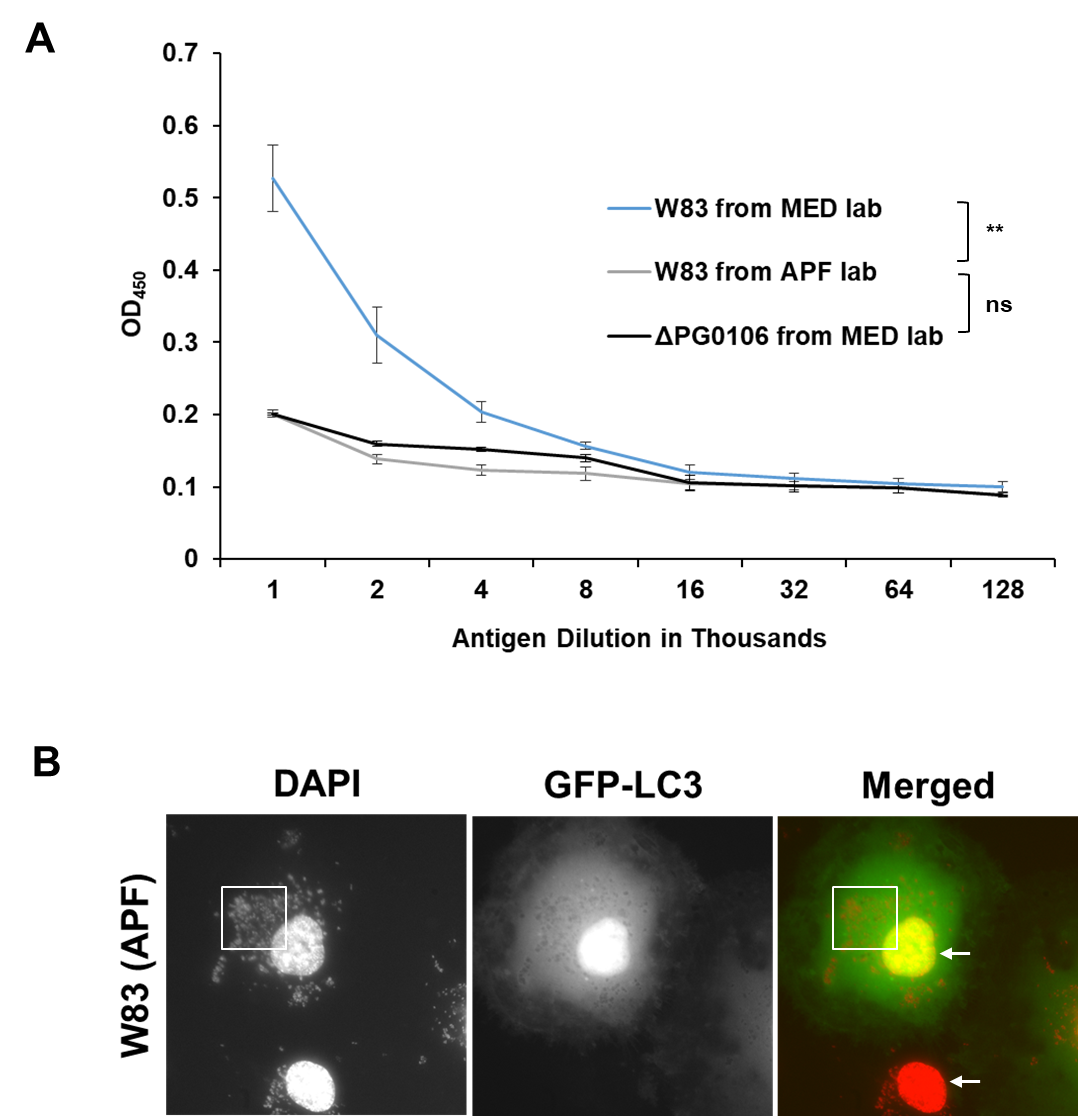
**

**Supplementary Figure 4.** Expression level of K-antigen capsule and intracellular trafficking in HCAEC of *P. gingivalis* strain W83 from the APF lab. **(A)** Enzyme-linked immunosorbent assays (ELISAs) were performed to detect K-capsular polysaccharide. Data represent the average of three biological replicates with error bars. The data of the antigen dilution 1/1000 were analyzed using the Student’s *t* test. ***p* < 0.01, ns *p* > 0.05. **(B)** Representative microscopic images of *P. gingivalis* strain W83 (red dots) with GFP-LC3 (green) at 2.5 hours post-inoculation. *P. gingivalis* cells (white boxes) and nuclei of HCAECs (white arrows) were stained with DAPI (pseudocolored red).

**
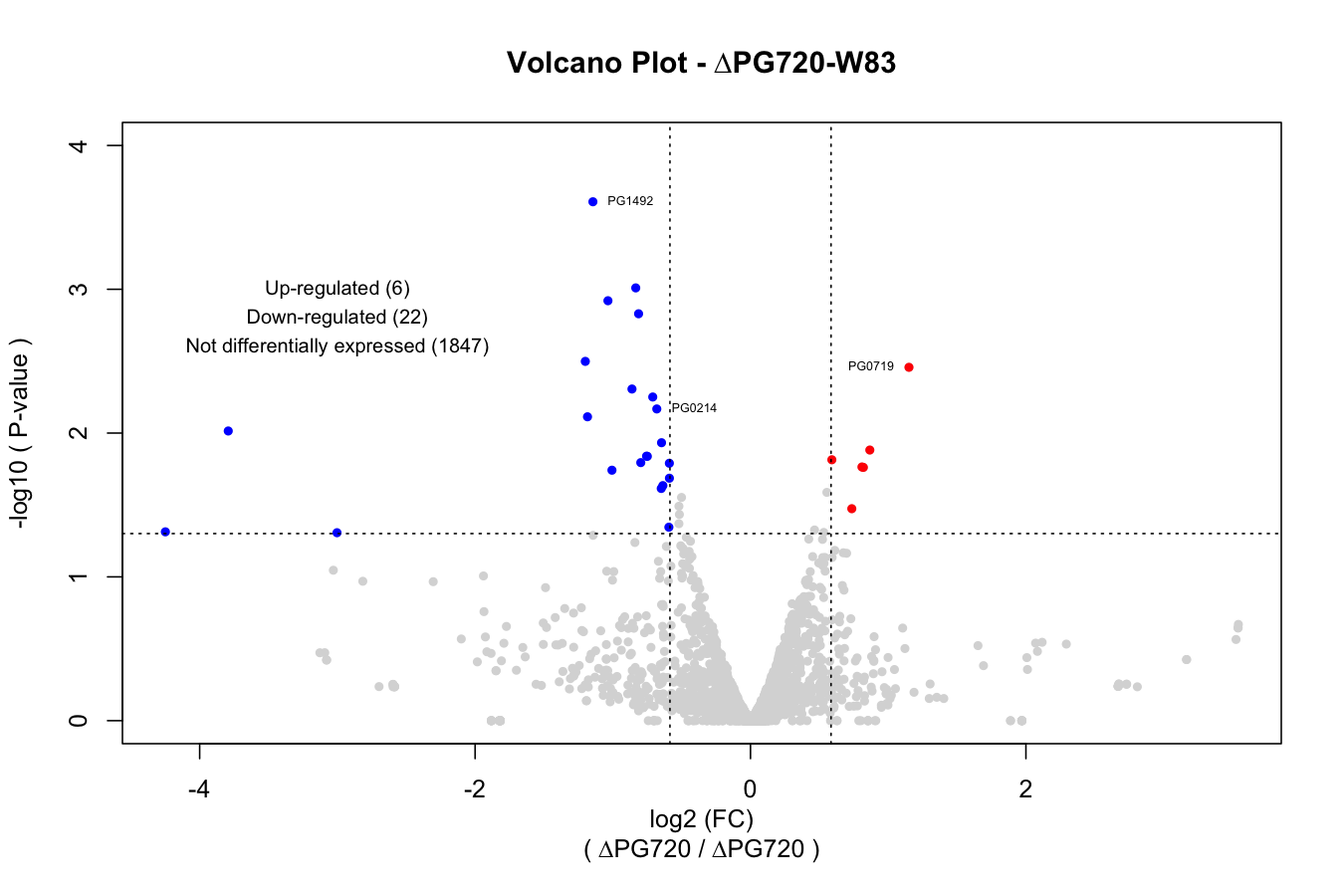
**

**Supplementary Figure 5**. A volcano plot shows the differentially expressed genes between ΔPG0720 mutant versus *P. gingivalis* parent strain W83. *Red dots* represent the significantly up-regulated genes and *blue dots* stand for the significantly down-regulated genes in ΔPG0720 mutant. Fold change > 1.5, *p*-value < 0.05.


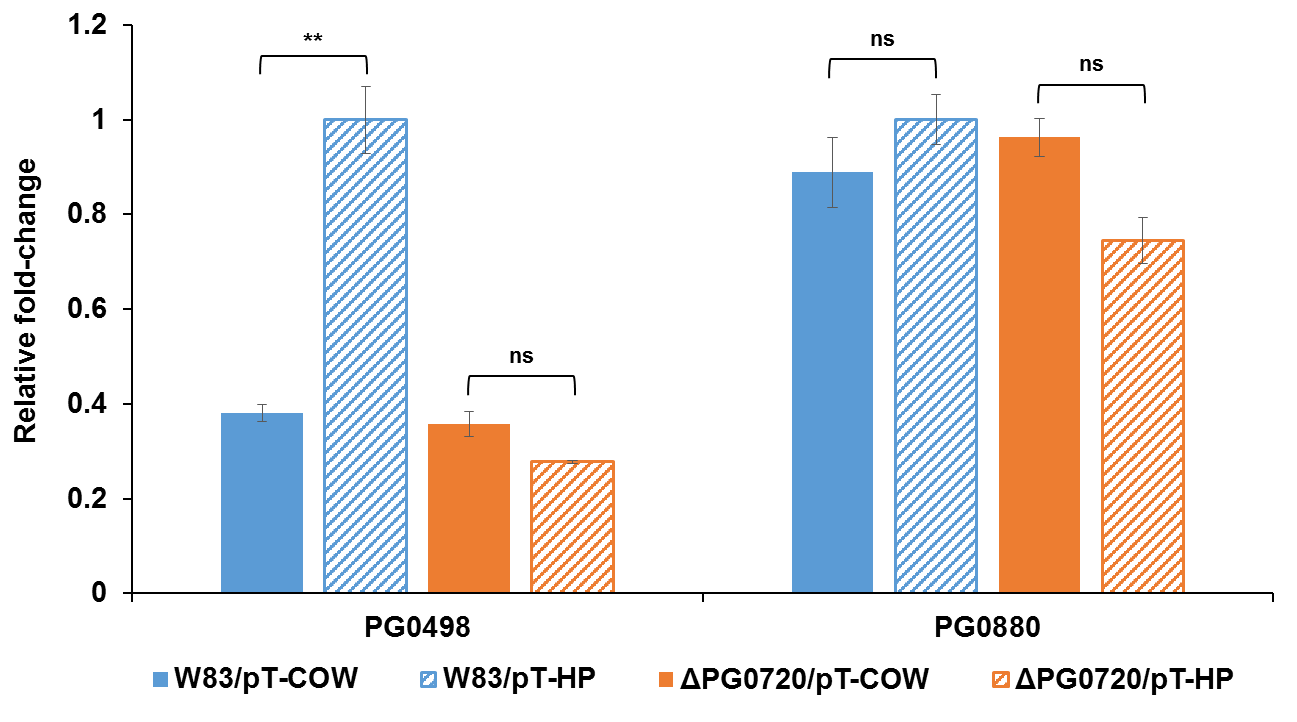


**Supplementary Figure 6.** qPCR to validate the effects of asSuGR overexpression on the expression levels of genes flanking other copies of the 77bpIR element on the chromosome. The relative transcript levels of genes flanking other copies of the 77bpIR element on the chromosome, including PG0498 and PG0880, were determined on the strain W83 and ΔPG0720 mutant harboring the empty plasmid (pT-COW) or pT-HP overexpresses the asSuGR. The results are presented as the relative levels (mean ± S.D. of triplicate determinations) compared with the transcript levels of the parent strain W83 harboring the plasmid pT-HP. The data were analyzed using the Student’s *t* test. ***p* < 0.01, ns *p* > 0.05.

**Supplementary Table 1. Primers used in this study**

| **Name** | **Sequence (5’-3’)** | **Purpose** |
| --- | --- | --- |
| pUC19_FW | GCGGGCCTCTTCGCTATTAC | Sequencing of *Δ*PG0720::Erm on plasmid |
| pUC19_RV | GATGCGGTATTTTCTCCTTAC | Sequencing of *Δ*PG0720::Erm on plasmid |
| PG0720_1000UP_FW | TTGTAAAACGACGGCCAGTGAATTCAAATATGTATCGTTTTTTTTCTCTCTC | Deletion of *Δ*PG0720::Erm |
| PG0720_1000UP_RV | AAGCTATCGGCTGGATAGCCCTGTTTGG | Deletion of *Δ*PG0720::Erm |
| PG0720_erm_FW | GGCTATCCAGCCGATAGCTTCCGCTATTG | Deletion of *Δ*PG0720::Erm |
| PG0720_erm_RV | CTCTTTCTATTCATCTTGACAACCACCC | Deletion of *Δ*PG0720::Erm |
| PG0720_1000DW_FW | GTCAAGATGAATAGAAAGAGAAGAAGATACTTGAAAC | Deletion of *Δ*PG0720::Erm |
| PG0720_1000DW_RV | CTATGACCATGATTACGCCAAGCTTAGCAAGACTTTGGCCAAAG | Deletion of *Δ*PG0720::Erm |
| PG0720_1100UP_FW | GGCTTCGTTTATATATCCGAAGGC | Sequencing of *Δ*PG0720::Erm |
| PG0720_1100DW_RV | GTATTCGCTATGCTGCAATGCATAG | Sequencing of *Δ*PG0720::Erm |
| pUC19_HindIII | AAGCTTGGCGTAATCATGG | Deletion of *Δ*PG0720::Erm |
| pUC19_EcoRI | GAATTCACTGGCCGTCGTTTTACAACG | Deletion of *Δ*PG0720::Erm |
| proPG0720_FW | acgcagtcaggcaccgtgtaAGAAGGTGAGGAAAAAAG | Cloning of PG0720 with their own promoter on pT-COW |
| proPG0720_RV | aggtgccgccggcttccattTCAAGTATCTTCTTCTCTTTC | Cloning of PG0720 with their own promoter on pT-COW |
| PG1138-F1 | GCTGCGAAAAAGTTCTGTCC | qRT-PCR of PG1138 |
| PG1138-R1 | GTATATCCCCCTCCCATTGC | qRT-PCR of PG1138 |
| PG1141-Forw | GGCAGCAGATTATGCCATCA | qRT-PCR of PG1141 |
| PG1141-Rev | GAGGCAAATCAGTGGCTACAA | qRT-PCR of PG1141 |
| PG0104_FW | CTTGGGACAAGCAGCTAAGG | qRT-PCR of PG0104 |
| PG0104_RV | TGTGTCGTTTTCTCCTGCAC | qRT-PCR of PG0104 |
| PG0106_FW | GGTTGACGCAGAGACAAACA | qRT-PCR of PG0106 |
| PG0106_RV | GCGCGAAAGATTGGCTATAA | qRT-PCR of PG0106 |
| PG0108_FW | TTGCCACTCGAGTAATGCAG | qRT-PCR of PG0108 |
| PG0108_RV | AGGATGACTTTGTCGGTTCG | qRT-PCR of PG0108 |
| PG0113_FW | AGGCCTTGGTGTTGGTAATG | qRT-PCR of PG0113 |
| PG0113_RV | TACTCCGAAATAGGCGTTGC | qRT-PCR of PG0113 |
| PG0118_FW | ACCAATTGGATCCCCAAAA | qRT-PCR of PG0118 |
| PG0118_RV | AGCAGGTTACGATGGAGCAT | qRT-PCR of PG0118 |
| PG0121_FW | GAAAAGGCCAACCTCACAAA | qRT-PCR of PG0121 |
| PG0121_RV | CGGTTTGAAACGAACAACCT | qRT-PCR of PG0121 |
| PG0720_FW | CAATGGGCTTACCCTTTTGA | qRT-PCR of PG0720 |
| PG0720_RV | TGTCCGGTACCAAAGACACA | qRT-PCR of PG0720 |
| asSuGR-Forw | GATTGGAGGAGCACAAGTGCTGTT | qRT-PCR of asSuGR |
| asSuGR-Rev | AGTGGAAAGATAGCTTTCCACCGG | qRT-PCR of asSuGR |
| PG0498_FW | GGCACCCGAACTGCATAC | qRT-PCR of PG0498 |
| PG0498_RV | CTGCATGAGGGGCAGTATATC | qRT-PCR of PG0498 |
| PG0880_FW | TAGACCAAGACGGGAACGAG | qRT-PCR of PG0880 |
| PG0880_RV | TCACTCCGATGATTTCGTAGC | qRT-PCR of PG0880 |
| asSuGRpro_FW | TGTCAGGGCGATCGCGTTTG | 154-bp (or 67-bp) region for EMSA |
| asSuGRpro_RV | TGGAATGATTCAGGACAATCG | 154-bp region for EMSA |
| PG0106pro_FW | GTCCTGAATCATTCCACTGG | 248-bp region for EMSA |
| PG0106pro_RV | ACACATCACAACAATATCTTC | 248-bp region for EMSA |
| PG0106_loop_RV | CCATATGTCAGAGATTCAAATC | 67-bp region for EMSA |
| 77bpIR2_FW | ATTCGCACTTGTTTGATAATTGAATGTAGCAATCATGAGATCTATAAGTGTGTAACGGTTCAAATGCGATTGT  CCTG | 77-bp region for EMSA |
| 77bpIR2_RV | CAGGACAATCGCATTTGAACCGTTACACACTTATAGATCTCATGATTGCTACATTCAATTATCAAACAAGTGCGAAT | 77-bp region for EMSA |
| 96bp_asSuGRpro_FW | ACCCCGGGGGTAGCCTGTCAG | 97-bp region for DNaseI footprinting |
| 96bp_asSuGRpro_RV | CAAACAAGTGCGAATCCATATG | 97-bp region for DNaseI footprinting |
| HP_Up_HindIII-F | GCGAAGCTTGGACACTAATACTCCCTGCT | Cloning of hairpin overexpression region on pT-COW |
| HP_Dn-BamHI-R | CGCGGATCCCATCCCTGAAATTTACACATC | Cloning of hairpin overexpression region on pT-COW |
| pSUMO_PG0720_FW | CGCGAACAGATTGGAGGTGGAAAAATCCTCATTATCGAAGATG | Cloning of PG0720 on pRham N-His SUMO |
| pSUMO_PG0720_RV | GTGGCGGCCGCTCTATTAAGTATCTTCTTCTCTTTCTATGAG | Cloning of PG0720 on pRham N-His SUMO |

**Supplementary Table 2. Strains and plasmids used in this study**

| **Strain (relevant genotype)** | **Source or reference** |
| --- | --- |
| ***P. gingivalis* strains** | |
| W83 (MED) | Christian Mouton, Laval University, Quebec City, Canada |
| W83 (APF) | SUNY-Buffalo collection^a^ |
| *Δ*PG0106::Erm (Em^r^) in strain W83 (MED) | Davey and Duncan (1) |
| *Δ*PG0720::Erm (Em^r^) in strain W83 (MED) | This study |
| ***E. coli* strain** | |
| NEB 5α | NEB |
| 10G | NEB |
| **Plasmids** | |
| pUC19 | NEB |
| pT-COW ([Ap^r^]^b^ Tc^r^) | Gardner *et al*. (2) |
| pT-PG0720 ([Ap^r^]^b^ Tc^r^) | This study |
| pRham N-His SUMO-PG0720 | This study |
| pT-HP ([Ap^r^]^b^ Tc^r^) | This study |

^a^SUNY-Buffalo = State University of New York at Buffalo.

^b^*E. coli* resistances in [parentheses].

**References**

1. Davey ME, Duncan MJ. 2006. Enhanced biofilm formation and loss of capsule synthesis: deletion of a putative glycosyltransferase in Porphyromonas gingivalis. J Bacteriol 188:5510-23.

2. Gardner RG, Russell JB, Wilson DB, Wang GR, Shoemaker NB. 1996. Use of a modified Bacteroides-Prevotella shuttle vector to transfer a reconstructed beta-1,4-D-endoglucanase gene into Bacteroides uniformis and Prevotella ruminicola B(1)4. Appl Environ Microbiol 62:196-202.
